# Efficacy of stability exercise-based treatment strategies for non-specific neck pain: a network meta-analysis

**DOI:** 10.3389/fphys.2026.1878997

**Published:** 2026-07-23

**Authors:** Xinyuan Li, Shaojuan Hu, Guanming Huang, Zhuoying Lin, Bin Guo

**Affiliations:** School of Physical Education and Sports Science, Hengyang Normal University, Hengyang, Hunan, China

**Keywords:** neck disability, network meta-analysis, non-specific neck pain, pain, range of motion, stability exercise

## Abstract

**Background:**

Non-specific neck pain (NSNP) is a common musculoskeletal disorder. Stability exercise is widely used in conservative management, but the relative efficacy of different exercise strategies remains unclear. This network meta-analysis evaluated stability exercise-based strategies for NSNP.

**Methods:**

PubMed, Embase, Cochrane Library, and Web of Science were searched from inception to November 24, 2025. Randomized controlled trials (RCTs) involving adults with NSNP were included. Risk of bias was assessed using RoB 2.0. A Bayesian network meta-analysis was conducted, and continuous outcomes were expressed as mean differences (MDs) with 95% credible intervals (95% CrIs). Global consistency and convergence were assessed using ΔDIC and PSRF, respectively. The certainty of evidence for key comparisons was evaluated using CINeMA.

**Results:**

Eighteen RCTs involving 950 participants were included; 10 studies were judged as low risk of bias, 4 as having some concerns, and 4 as high risk. Global consistency and convergence were acceptable (all ΔDIC < 5; PSRF = 1), with low-to-moderate heterogeneity across outcomes. For pain, several stability exercise strategies significantly reduced visual analog scale (VAS) scores compared with self-organized exercise (SOE), with shoulder resistance training combined with chest flexibility training (SRT+CFT) showing the greatest estimated reduction (MD = −8.16, 95% CrI: −13.04 to −3.27). For function, no intervention showed a significant advantage for the neck disability index (NDI), although neck resistance training combined with core resistance training (NRT+CRT) had the highest SUCRA value (MD = −27.43, 95% CrI: −58.28 to 3.90). For range of motion (ROM), neck flexibility training combined with NRT (NFT+NRT) had the highest SUCRA values for neck flexion, extension, bilateral lateral flexion, and bilateral rotation. The certainty of evidence was generally low or very low, with few moderate-certainty estimates.

**Conclusion:**

Stability exercise-based strategies showed outcome-specific effects in NSNP. NRT, SRT, CRT, NFT, and their combinations may be more beneficial for pain relief and ROM improvement, whereas no clear superiority was found for functional recovery. These findings should be interpreted cautiously because certainty was generally low or very low and several treatment nodes were supported by limited evidence.

**Systematic review registration:**

https://www.crd.york.ac.uk/PROSPERO/view/, identifier CRD420251243575.

## Introduction

Non-specific neck pain (NSNP) represents one of the most frequent types of musculoskeletal pain (Cohen, 2015). It has become a significant public health concern due to its high prevalence, recurrence, and disabling burden. Recent evidence based on GBD implied that about 203 million people worldwide were afflicted by neck pain in 2020. The disease burden is higher in women than in men (approximately 2890/100,000 vs. 2000/100,000). The burden peaked in individuals aged between 45 and 74 years, when their working and family responsibilities are the most burdensome (GBD 2021 Neck Pain Collaborators, 2024). Furthermore, it is estimated that neck pain cases may reach 269 million by 2050, a 32.5% increase from 2020 (GBD 2021 Neck Pain Collaborators, 2024; Wu et al., 2025). Consequently, the prevention and treatment of NSNP is urgent.

Current conservative treatments for NSNP include health education, manual therapy, physical agents, and exercise therapy. Systematic reviews have indicated that various exercise modalities can generally reduce pain and improve function (Rasmussen-Barr et al., 2023), but there are still differences in the effect sizes and stability across different types of training (Mueller et al., 2023). Because NSNP is often accompanied by decreased endurance of deep neck muscles, abnormal activation of muscles, and impaired neuromuscular control, stability exercise for activating deep muscles, improving motor control, and enhancing proprioception is increasingly used to manage NSNP in recent years. Related evidence also suggests that stability exercise can improve pain, muscle recruitment patterns and local function (Tsiringakis et al., 2020; Iqbal et al., 2021; Dirito et al., 2024). Other stability training strategies have also been reported to have clinical benefits in relieving pain and improving function (Aydoğmuş et al., 2022; Gumuscu et al., 2023; Chaiyawijit and Kanlayanaphotporn, 2024). Additionally, scapular-related training may alleviate pain by improving load distribution in the neck-shoulder kinetic chain, but the evidence for functional outcomes and certain kinematic parameters remain somewhat inconsistent (Javdaneh et al., 2021; Chen et al., 2024).

Although stability exercise is widely used, the optimal stability exercise strategy is inconclusive. Existing original RCTs mostly compare a single stability exercise strategy with routine care or general exercise, and direct head-to-head evidence between different stability exercise modalities is lacking (Iqbal et al., 2021; Aydoğmuş et al., 2022; Gumuscu et al., 2023; Chaiyawijit and Kanlayanaphotporn, 2024). Meanwhile, previous systematic reviews often report stability exercise in general terms but do not distinguish between body regions or training nature. In contrast, In this review, stability training is operationally defined as a training prescription framework based on components, rather than a single training method. Specifically, interventions are categorized according to primary anatomical areas (neck, shoulder girdle, chest, or core) and primary training objectives (flexibility or resistance), as these components are often used in combination in clinical practice to improve regional control, coordination stability, and load distribution within the neck, shoulder, chest, and core system. Pre-defined operational definitions and coding rules for all intervention nodes are detailed in **Supplementary Table 1**. Notably, the absolute burden of neck pain increased significantly from 1990 to 2019 and was predicted to continue to rise until 2044 (Cheng et al., 2024). Hence, determining which stability exercise is more effective is significant.

Network meta-analysis (NMA) is able to integrate direct and indirect comparisons within a network framework, and simultaneously compare and rank multiple protocols in the absence of head-to-head trials, thereby providing high-quality evidence for the selection of clinical prescriptions (Hutton et al., 2015; Salanti et al., 2022; Curteis et al., 2025). This NMA intended to systematically compare and rank the efficacy of various stability exercise methods on pain and function in individuals with NSNP, aiming to offer high-quality evidence for optimizing rehabilitation decisions, reducing the disability burden, and improving patient outcomes in clinical settings.

## Methods

This NMA was executed as per the Preferred Reporting Items for Systematic Reviews and Meta-Analyses (PRISMA) for Network Meta-Analyses Extension (Page et al., 2021). The study protocol was registered on the International Prospective Register of Systematic Reviews (PROSPERO) (CRD420251243575).

### Search strategy

PubMed, Embase, Cochrane Library, and Web of Science databases were retrieved from database inception to November 24, 2025. The search terms were designed by combining subject terms and free terms. The medical subject terms used included “Neck Pain,” “Non-Specific,” “Resistance Training,” “Muscle Stretching Exercises,” and “Core Stability Training.” References in related articles and grey literature were also manually retrieved to identify other eligible articles. Specific search strategies are detailed in **Supplementary Table 2**.

### Inclusion and exclusion criteria

Studies meeting the following criteria were included: (i) Participants: Adult patients diagnosed with NSNP (≥ 18 years old). Specific causes were ruled out (such as radiculopathy/spinal cord disease, fracture, tumor, infection, and inflammatory diseases). The following populations were excluded: (a) those with a history of cervical spine surgery or recent severe trauma; (b) those with clearly defined predominant nerve root symptoms (e.g., radiating pain with neurological signs) or spinal cord compression; (c) those with systemic diseases (e.g., rheumatoid arthritis) or pregnancy that significantly affect the safety of exercises; and (d) those with a primary diagnosis of a specific subtype with a significantly different mechanism (e.g., whiplash-associated disorder or specific cervicogenic headache). (ii) Intervention methods included neck flexibility training (NFT), neck resistance training (NRT), shoulder flexibility training (SFT), shoulder resistance training (SRT), chest flexibility training (CFT), core resistance training (CRT), and their combinations. The control group used physical therapy (PT) or self-organized exercises (SOE). (iii) Study type: randomized controlled trial (RCT) (Rasmussen-Barr et al., 2023). Outcomes: Visual Analog Scale (VAS), neck disability index (NDI), and range of motion (ROM).

The following studies were excluded: (i) animal or cell experiments, case reports, scientific experimental plans, reviews, letters, editorials, conference papers, etc.; (ii) research with missing or seriously erroneous research data; (iii) duplicate publications; (iv) full texts not found; (v) studies with duplicate participants.

### Data extraction

The retrieved records were imported into EndNote. Two investigators independently screened the titles and abstracts as per the eligibility criteria. Subsequently, the full texts were read to select eligible studies. Dissents, if any, were discussed between them or assessed again as per the suggestions of a third investigator. Data extraction was implemented independently by two investigators employing a pre-defined spreadsheet. The extracted information encompassed first author, publication year, country, randomization and blinding design, intervention and control measures, treatment duration, basic information of study subjects, and outcome indicators.Two reviewers independently coded the intervention nodes based on pre-defined operational definitions (based on the target region and main training goal) (**Supplementary Table 1**).

### Quality assessment

Two investigators independently adopted the Cochrane Risk of Bias Tool (ROB2.0) (Sterne et al., 2019) to assess the quality of the included articles. The ROB 2.0 tool includes five aspects: the generation of random sequences, allocation concealment, blinding, missing data, and selective reporting. Each domain was graded as a high, moderate, or low risk of bias. If all five domains in an article were graded to have a low risk of bias, or only one domain was graded to have a moderate risk and the rest had a low risk, the risk of bias in the article was considered low. If the risk of bias in four or more of the five domains in an article was moderate, or any one of them was deemed to have a high risk, the risk of bias in the article was considered high; otherwise, the article was deemed to have a moderate risk. Any dissents were addressed by a third investigator.

### Statistical analysis

All outcome measures were continuous variables and were expressed as the mean difference (MD) and 95% credible interval (CrI). The Markov chain-Monte Carlo method was employed to construct a Bayesian-based NMA model, which was iterated to examine the relative effectiveness of distinct treatment regimens. During the testing process, the model chain value was set at 4, the annealing value at 10000, the iteration value at 50000, the step size for each test at 10, and the initial value at 2.5. This process intended to obtain the posterior distribution. For NMA, three basic assumptions must be satisfied: transitivity, homogeneity, and consistency assumptions. When I² was less than 50%, the heterogeneity among included studies was considered acceptable, meeting the homogeneity hypothesis. The consistency of evidence was assessed utilizing the deviation information criterion (DIC) of the consistency and inconsistency models. The DIC results for the consistency and inconsistency models were close (ΔDIC < 5), implying good consistency between the direct and indirect evidence in the included studies. To support the transitivity hypothesis, we performed a descriptive analysis of the distribution of key potential moderating factors at the intervention nodes, including age, symptom duration, baseline pain severity, chronicity (chronic vs. subacute), and treatment duration. Model convergence was assessed using the potential scale reduction factor (PSRF), together with visual inspection of MCMC trace plots and density plots. A PSRF value between 1.00 and 1.05 was considered to indicate acceptable convergence. Detailed convergence diagnostics are provided in **Supplementary Figure 1**. A network structure was developed, where nodes represented interventions and lines represented head-to-head comparisons between interventions. The numeric treatment codes used in the network meta-analysis are provided in **Supplementary Table 3**. The area under the cumulative rank curve (SUCRA) was calculated to describe the relative rank probability of the interventions. The SUCRA value should be interpreted in conjunction with the effect estimate and the 95% confidence interval (CrI), rather than as evidence of superiority. Exploratory meta-regression analyses were performed for outcomes with sufficient data to examine the potential influence of treatment duration and sample size. The detailed results are presented in **Supplementary Tables 4**–**S7**. Funnel plots are employed to examine potential publication bias. Statistical analyses were implemented utilizing R (version 4.5.1) and STATA (version 18.0).

### Certainty assessment

The Confidence in Network Meta-Analysis (CINeMA) tool (available at https://cinema.ispm.unibe.ch/) was used to assess the quality of evidence for all pairwise comparisons. This tool is designed to evaluate the reliability of network meta-analysis results (Nikolakopoulou et al., 2020). It considers six key areas: within-study bias (risk of bias), between-study bias (publication or reporting bias), indirectness, imprecision, heterogeneity, and inconsistency. Each area was categorized into three levels based on the degree of bias: no concern (no downgrade), concern (downgraded one level), and significant concern (downgraded two levels). Ultimately, the evidence for each pairwise comparison was rated as “high,” “moderate,” “low,” or “very low.”

## Results

### Literature retrieval and screening

**Figure 1:**
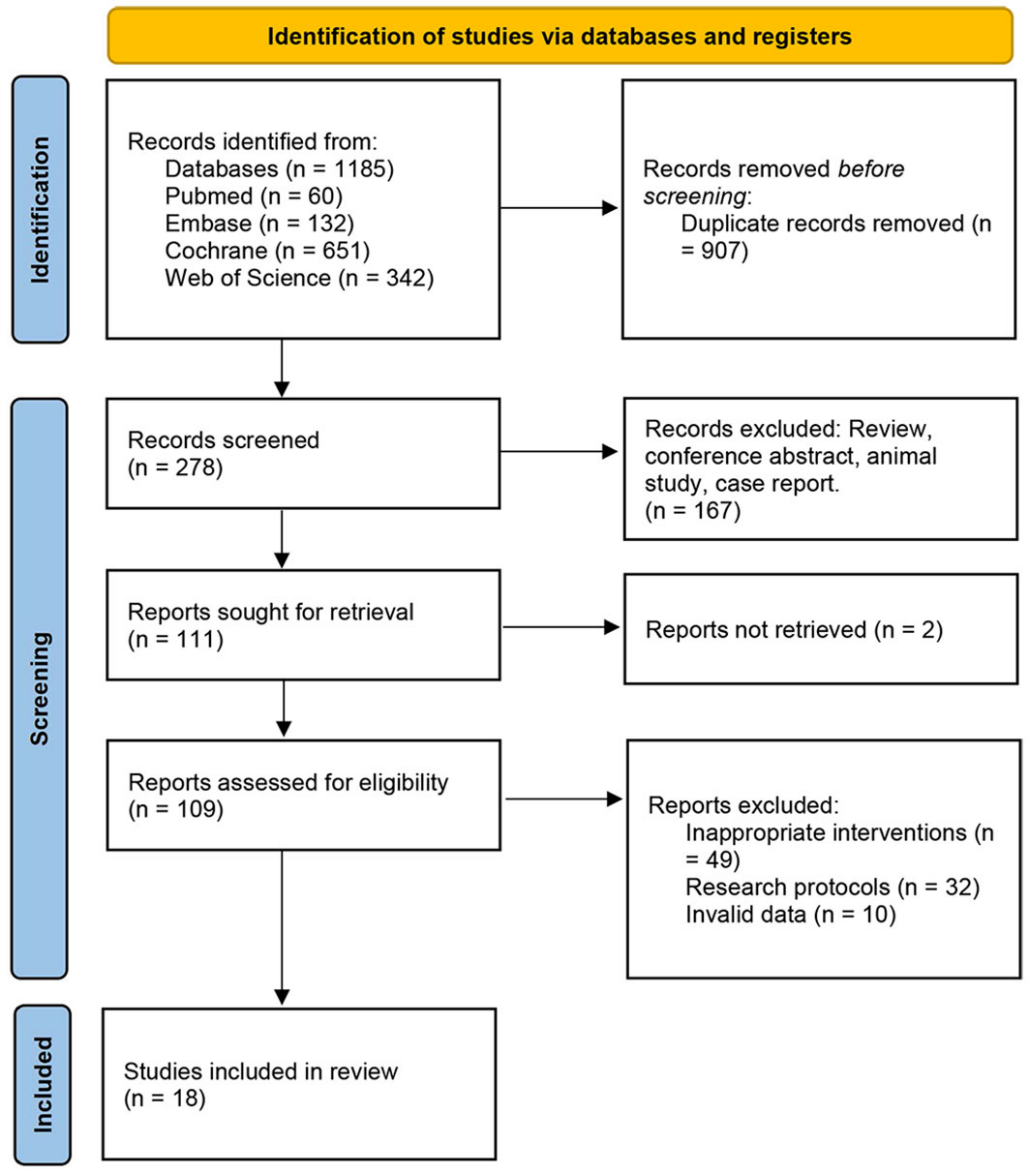
Literature search and screening process.

Initially, 1185 publications were retrieved. Then, 907 duplicates were removed. After screening the titles and abstracts, 278 publications were eliminated. The full texts of the remaining publications were then checked. Finally, 18 articles were incorporated into this NMA. The specific screening process is depicted in **Figure 1**.

### Basic characteristics and quality assessment of included studies

The 18 included studies came from 10 countries (China, Turkey, Iran, Pakistan, Egypt, Nigeria, Spain, Korea, Norway, and India), involving a total of 950 patients. 38.4% of these patients were male, and 61.6% were female. The age of participants ranged from 20 to 78 years old. The involved interventions included neck flexibility training (NFT), neck resistance training (NRT), shoulder flexibility training (SFT), shoulder resistance training (SRT), chest flexibility training (CFT), core resistance training (CRT), NFT+NRT, NFT+NRT+SRT, SRT+NFT, SRT+CFT, NRT+CRT, NFT+SFT, NRT+CRT+SRT, and NFT+SFT+NRT. Basic features of the included publications are depicted in **Table 1**.

**Table 1:** Basic features of eligible studies.

Authors	Year	Study design	Area	Interventions	Sample size	Ages	Treatment duration	Outcomes	Symptom duration	Baseline pain severity	Chronic vs subacute
Demir, O et al.	2023	RCT	Turkey	NFT+NRT	15	30 ± 7	6W	VAS, NDI, ROM	≥3M	VAS:5.8 ± 1.57	Chronic
				NRT	15	33 ± 11				VAS:6.33 ± 1.68	
Batool, A et al.	2024	RCT	Pakistan	NFT	30	30.33 ± 6.68	3W	VAS, NDI, ROM	3W to 6M	VAS:3.5–7.4	Chronic
				NRT	30	29.83 ± 6.1					
Hassan, W et al.	2016	RCT	Pakistan	NRT	19	/	3W	VAS, NDI, ROM	≥7W	NDI (50)≥15	Chronic
				NFT+NRT	21						
Zhang, Y et al.	2024	RCT	China	NFT+NRT	35	28.95 ± 7.86	12M	NDI, VAS	≥3M	NDI (100)≥50 and VAS≥4	Chronic
				NFT	35	29.46 ± 8.33					
Amir Letaftakar et al.	2020	RCT	Iran	NFT+NRT+SRT	24	34.27 ± 2.72	8W	VAS, NDI	≥3M	NDI (100):37.1 ± 4.5,VAS:5.9 ± 0.9	Chronic
				SOE	24	34.60 ± 3.30					
Doaa I. A. et al.	2024	RCT	Egypt	NFT	38	42.05 ± 8.93	6W	VAS, ROM	-	NPRS:5.8 ± 1.3	Chronic
				NFT+NRT	38	40.11 ± 7.00				NPRS:5.9 ± 1	
Kaka, B. et al.	2018	RCT	Nigeria	NFT+SFT	30	46.8 ± 12.4	8W	VAS	≥6W	VAS:7.56 ± 1.65	Chronic
				NRT	31	45.1 ± 13.4				VAS:7.03 ± 1.57	
				NFT+SFT+NRT	28	48.6 ± 11.6				VAS:6.80 ± 1.57	
Ximei S. et al.	2024	RCT	China	SRT	10	/	6W	VAS, NDI, ROM	≥3M	VAS≥3	Chronic
				SRT+NFT	10						
				SRT+CFT	10						
Hernandez Lucas, P. et al.	2023	RCT	Spain	NRT	18	51.9 ± 7.8	8W	VAS	≥3M	VAS:3-7	Chronic
				SOE	17	51.4 ± 11.3					
Soroush, S. et al.	2022	RCT	Iran	NRT	33	72.06 ± 5.91	12W	VAS, NDI	≥3M	VAS≥3	Chronic
				CRT	33	72.36 ± 5.30					
				NRT+CRT	36	69.39 ± 5.20					
Khan, M A et al.	2024	RCT	Pakistan	SRT	13	24.76 ± 4.53	3W	VAS, NDI, ROM	≥3M	NPRS:6.38 ± 1.04	Chronic
				NFT+SFT	13	27.76 ± 4.62				NPRS:6.46 ± 1.12	
SinHo Chung and Yeon Gyu Jeong	2018	RCT	Korea	NFT	22	34.27 ± 8.74	8W	VAS, NDI, ROM	≥3M	VAS:4.85 ± 1.56	Chronic
				NRT	19	37.01 ± 10.24				VAS:5.26 ± 0.99	
Abdel Aziem, A. A. and Draz, A. H.	2016	RCT	Egypt	PT	20	48.5 ± 7.8	4W	VAS, NDI, ROM	≥6W	VAS:6.85 ± 1.09	Chronic
				NRT+CRT+SRT	20	47.9 ± 6.8				VAS:6.40 ± 1.10	
				NRT	20	50.1 ± 4.7				VAS:6.70 ± 0.98	
Mendes Fernandes, T. et al.	2023	RCT	Portugal/Spain	PT	25	47.84 ± 8.86	4W	VAS, NDI, ROM	≥12W	Pain Intensity≥2	Chronic
				NFT+NRT+SRT	25	53.80 ± 7.74					
Iversen, V. M. et al.	2018	RCT	Norway	NRT+CRT+SRT	29	44.6 ± 8.1	12W	NDI, VAS	≥3M	neck pain (the last 2 weeks)≥4	Chronic
				SOE	30	48.2 ± 10.6					
Ashtiani, AA. et al.	2014	RCT	Iran	NFT	25	34.08 ± 9.48	8W	VAS, NDI	≥12W	VAS:7.16 ± 1.57	Chronic
				NRT	25	36.24 ± 10.05				VAS:7.12 ± 1.67	
Parisa, GH. et al.	2016	RCT	Iran	NFT	22	26.09 ± 2.42	8W	VAS, NDI	≥12W	VAS:6.90 ± 1.47	Chronic
				NFT+NRT	22	26.09 ± 2.84				VAS:6.45 ± 1.22	
Rajalaxmi, V. et al.	2020	RCT	India	NRT	20	/	12W	VAS, NDI	-	VAS:6.6 ± 0.48	Chronic
				NRT+CRT	20	/				VAS:6.4 ± 0.66	

VAS, Visual Analog Scale; NDI, Neck Disability Index; ROM, Range of Motion; NFT, Neck Flexibility Training; NRT, Neck Resistance Training; SFT, Shoulder Flexibility Training; SRT, Shoulder Resistance Training; CFT, Chest Flexibility Training; CRT, Core Resistance Training.

**Figure 2:**
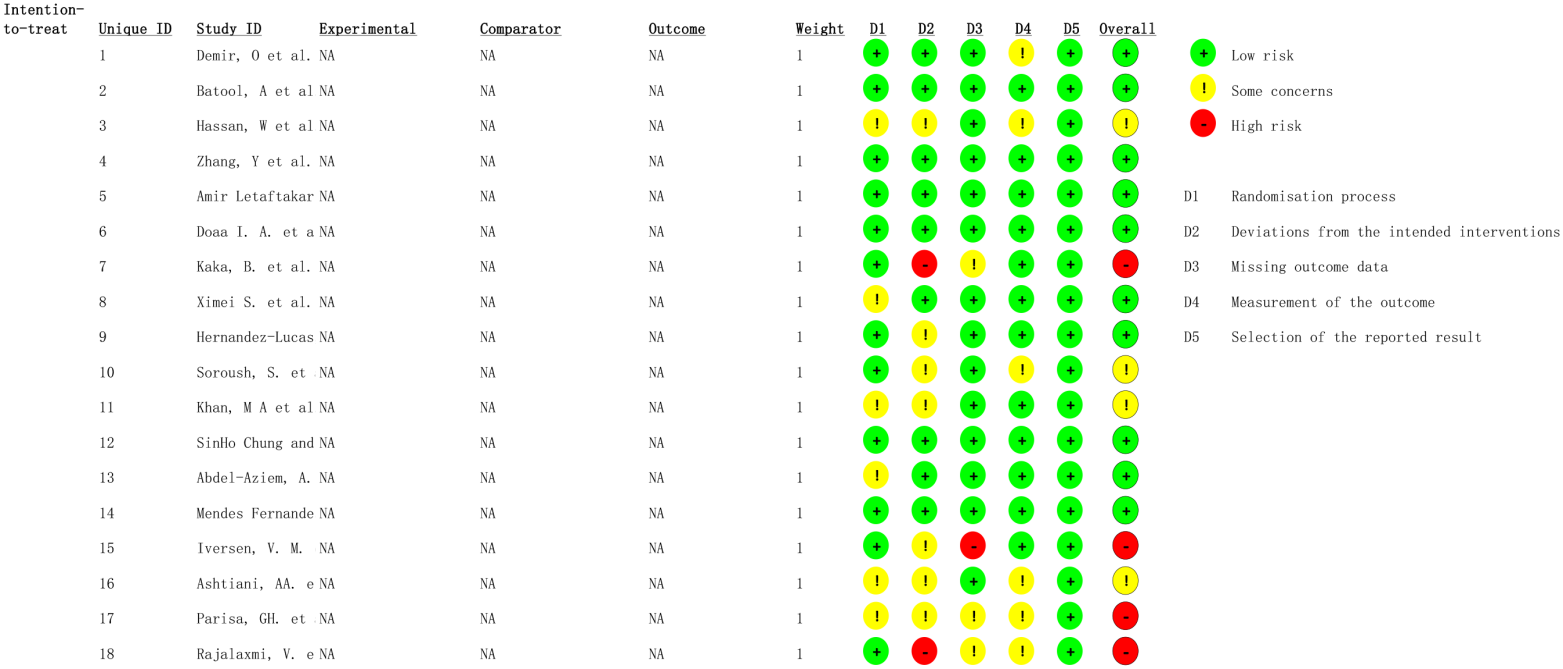
Quality assessment results of the included studies.

Most articles exhibited a low risk of bias. Overall, 10 studies were rated as low risk; 4 studies were rated as having some concerns; and 4 studies were rated as high risk. Four articles were deemed to have a moderate risk because they did not sufficiently report the randomization process, elucidate the use of intention-to-treat protocols, or blind the assessment of subjective outcome indicators. Four articles were considered to have a high risk owing to intervention implementers knowing the group assignments, deviations from the trial environment potentially affecting the outcomes, or potentially non-randomized missing data, because there was no evidence that missing data were unlikely to cause outcome bias, and the reason for missing data might be related to the true outcomes. The risk of bias assessment results of the eligible articles are illustrated in **Figure 2**.

### Results of NMA

#### Network relationship diagram

**Figure 3:**
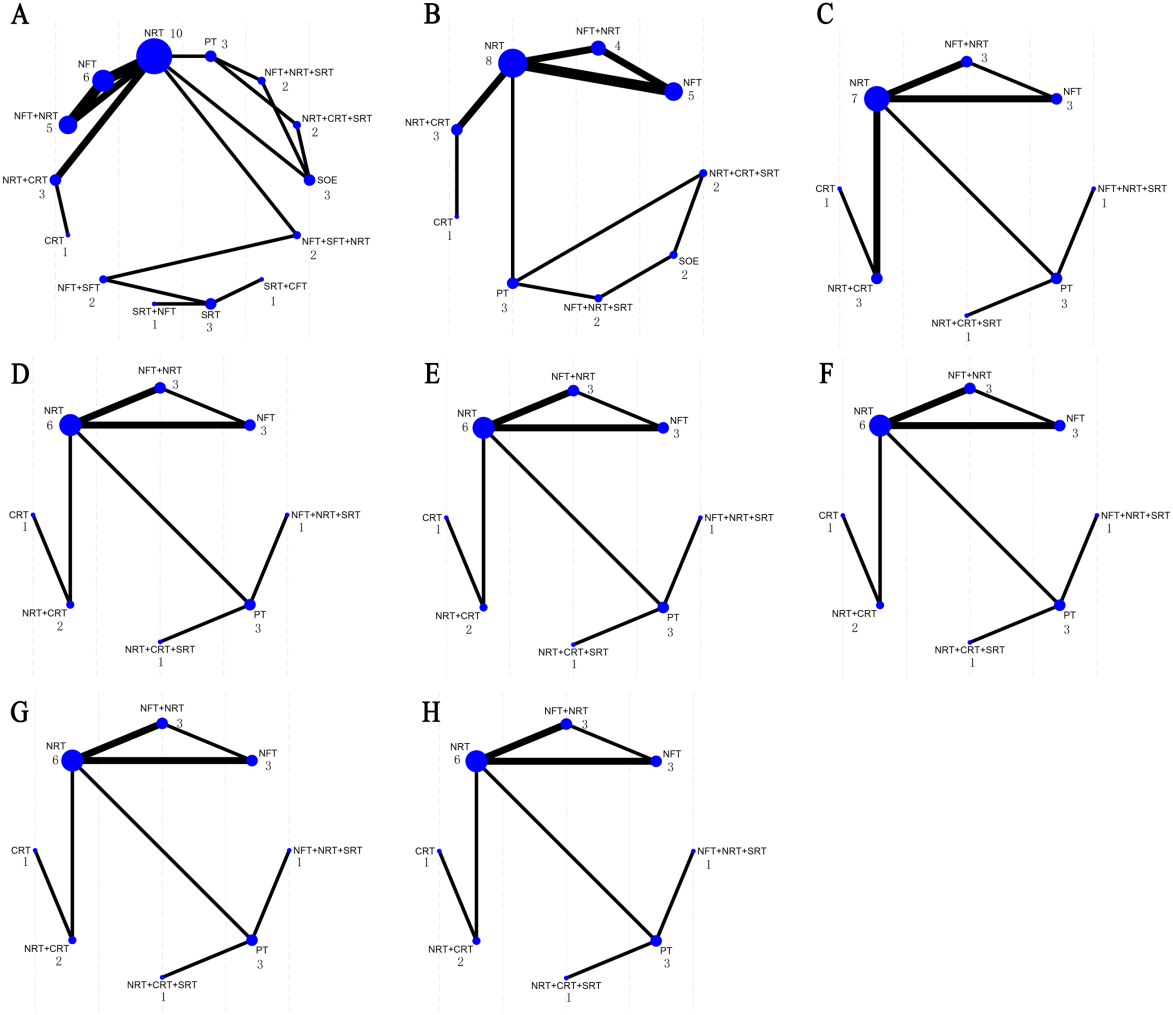
Network diagram of each outcome indicator. Each node denotes an intervention. The size of the node is proportional to the number of studies related to the intervention. The lines connecting the nodes denote a direct head-to-head comparison between the two interventions. The thickness of the lines is proportional to the number of studies. [**(A)** VAS; **(B)** NDI; **(C)** Neck flexion ROM; **(D)** Neck extension ROM; **(E)** Neck left lateral flexion ROM; **(F)** Neck right lateral flexion ROM; **(G)** Neck left rotation ROM; **(H)** Neck right rotation ROM].

In the network diagram, each node represents an intervention. The size of a node is proportional to the number of articles evaluating that intervention, and the thickness of a connecting line is proportional to the number of direct comparative articles between two interventions (**Figure 3**). The differences in DIC between the consistency and inconsistency models were less than 5 for all outcomes (VAS, NDI, neck flexion ROM, neck extension ROM, neck left lateral flexion ROM, neck right lateral flexion ROM, neck left rotation ROM, and neck right rotation ROM), indicating that the consistency model was acceptable for these outcomes (**Table 2**). Model convergence was satisfactory: PSRF values were equal to 1 for all outcomes (**Supplementary Figure 1**), and visual inspection of MCMC trace and density plots showed good convergence (**Supplementary Figure 2**). Node-splitting analysis revealed potential local inconsistency in a few comparisons, although most estimable comparisons showed no significant disagreement between direct and indirect evidence (**Supplementary Figure 3**).

**Table 2:** Degree of fitting of NMA model and heterogeneity analysis results.

	Dbar	pD	DIC		ΔDIC	I^2^	
			consistency	ume		consistency	ume
VAS	43.777	42.819	86.596	87.250	0.654	2%	2%
NDI	30.121	29.440	59.562	59.621	0.059	4%	4%
Neck Flexion ROM	41.444	18.011	59.455	58.730	0.725	49%	47%
Neck Extension ROM	23.984	17.008	40.992	42.341	1.349	21%	22%
Neck Left lateral flexion ROM	22.180	16.985	39.166	40.880	1.714	14%	17%
Neck Right lateral flexion ROM	23.406	17.004	40.410	41.626	1.216	19%	20%
Neck Left rotation ROM	23.086	17.029	40.115	41.903	1.788	18%	21%
Neck Right rotation ROM	30.146	18.003	46.313	48.149	1.836	35%	37%

Dbar, posterior mean residual deviance; pD, effective number of parameters; DIC, deviance information criterion; ΔDIC, absolute difference in DIC between the consistency and inconsistency models; I², heterogeneity statistic.

### The results of each outcome

#### Primary outcomes

##### VAS

**Figure 4:**
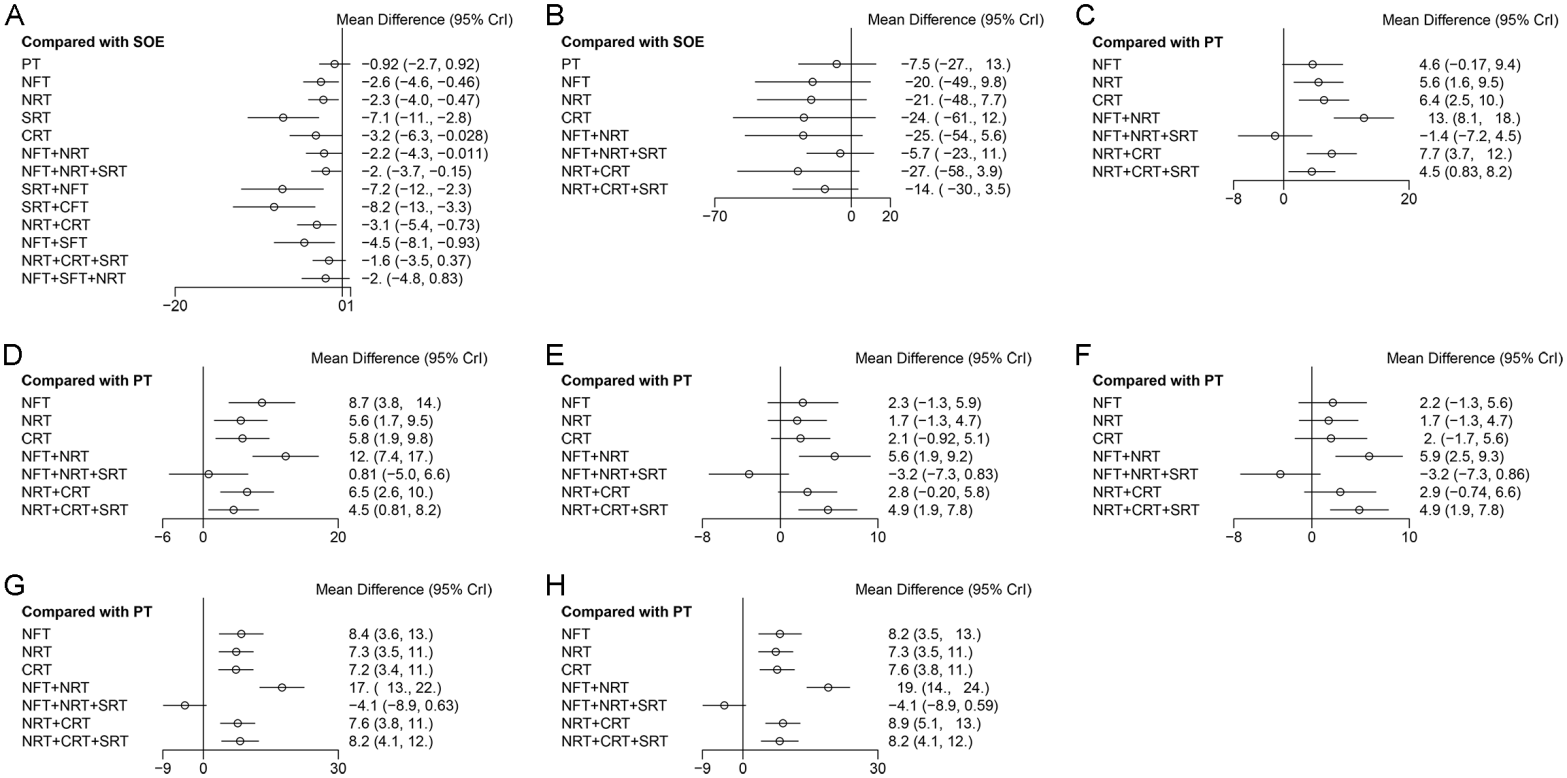
Forest plot of network meta-analysis for each outcome indicator. The figure shows the effect size and 95% credible interval (95% CrI) of each intervention vs the control, used to compare the relative efficacy of different stability exercise strategies on each outcome indicator. [**(A)** VAS; **(B)** NDI; **(C)** Neck flexion ROM; **(D)** Neck extension ROM; **(E)** Neck left lateral flexion ROM; **(F)** Neck right lateral flexion ROM; **(G)** Neck left rotation ROM; **(H)** Neck right rotation ROM].

A total of 18 studies reported VAS, including 898 patients. The NMA results indicated that, relative to SOE, NFT, NRT, SRT, CRT, NFT+NRT, NFT+NRT+SRT, SRT+NFT, SRT+CFT, and NRT+CRT significantly reduced VAS in patients with NSNP. Detailed pairwise comparisons are presented in **Table 3** (SOE vs NFT: MD = −2.56, 95%CrI: −4.63, −0.46; SOE vs NRT: MD = −2.27, 95%CrI: −4.04, −0.47; SOE vs SRT: MD = −7.07, 95%CrI: −11.28, −2.83; SOE vs CRT: MD = −3.16, 95%CrI: −6.26, −0.03; SOE vs NFT+NRT: MD = −2.18, 95%CrI: −4.31, −0.01; SOE vs NFT+NRT+SRT: MD = −1.95, 95%CrI: −3.68, −0.15; SOE vs SRT+NFT: MD = −7.17, 95%CrI: −12.06, −2.27; SOE vs SRT+CFT: MD = −8.16, 95%CrI: −13.04, −3.27; SOE vs NRT+CRT: MD = −3.05, 95%CrI: −5.36, −0.73; SOE vs NFT+SFT: MD = −4.54, 95%CrI: −8.13, −0.93). The forest plot is illustrated in **Figure 4A**.

**Table 3:** League table for VAS.

**SOE**													
-0.92 (-2.72, 0.92)	**PT**												
**-2.56** **(-4.63, -0.46)**	-1.64 (-3.74, 0.45)	**NFT**											
**-2.27 (-4.04,** **-0.47)**	-1.34 (-3.13, 0.44)	0.29 (-0.79, 1.37)	**NRT**										
**-7.07 (-11.28, -2.83)**	**-6.15** **(-10.38, -1.93)**	**-4.52 (-8.49,** **-0.53)**	**-4.81 (-8.64,** **-0.96)**	**SRT**									
**-3.16 (-6.26,** **-0.03)**	-2.24 (-5.38, 0.88)	-0.6 (-3.38, 2.19)	-0.89 (-3.47, 1.67)	3.91 (-0.73, 8.53)	**CRT**								
**-2.18 (-4.31, -0.01)**	-1.26 (-3.39, 0.89)	0.38 (-0.68, 1.46)	0.09 (-1.1, 1.29)	**4.89 (0.89,** **8.91)**	0.99 (-1.84, 3.82)	**NFT+NRT**							
**-1.95 (-3.68, -0.15)**	-1.03 (-2.81, 0.78)	0.61 (-1.79, 3.05)	0.31 (-1.84, 2.5)	**5.12 (0.73,** **9.54)**	1.21 (-2.14, 4.6)	0.23 (-2.23, 2.72)	**NFT+NRT+ SRT**						
**-7.17 (-12.06, -2.27)**	**-6.25 (-11.16, -1.38)**	-4.61 (-9.33, 0.08)	**-4.91 (-9.5,** **-0.35)**	-0.1 (-2.63, 2.39)	-4.02 (-9.28, 1.25)	**-5.01 (-9.73,** **-0.28)**	**-5.22 (-10.29, -0.19)**	**SRT+NFT**					
**-8.16 (-13.04, -3.27)**	**-7.24 (-12.13, -2.35)**	**-5.61 (-10.28, -0.91)**	**-5.9 (-10.46, -1.34)**	-1.09 (-3.55, 1.36)	-5.01 (-10.24, 0.24)	**-5.99 (-10.7,** **-1.27)**	**-6.21 (-11.26, -1.18)**	-0.99 (-4.49, 2.53)	**SRT+CFT**				
**-3.05 (-5.36,** **-0.73)**	-2.13 (-4.48, 0.17)	-0.49 (-2.34, 1.34)	-0.78 (-2.29, 0.69)	4.02 (-0.12, 8.12)	0.11 (-1.98, 2.2)	-0.87 (-2.8, 1.02)	-1.1 (-3.76, 1.5)	4.12 (-0.68, 8.93)	**5.11 (0.29,** **9.89)**	**NRT+CRT**			
**-4.54 (-8.13,** **-0.93)**	**-3.62 (-7.22,** **-0.02)**	-1.99 (-5.3, 1.32)	-2.28 (-5.41, 0.85)	**2.53 (0.32,** **4.74)**	-1.39 (-5.43, 2.67)	-2.37 (-5.73, 0.98)	-2.6 (-6.42, 1.2)	2.63 (-0.68, 5.99)	**3.62 (0.32,** **6.93)**	-1.5 (-4.95, 2)	**NFT+SFT**		
-1.59 (-3.51, 0.37)	-0.67 (-2.48, 1.16)	0.97 (-1.52, 3.49)	0.67 (-1.56, 2.95)	**5.48 (1.06,** **9.93)**	1.57 (-1.81, 5.01)	0.58 (-1.96, 3.15)	0.35 (-1.89, 2.61)	**5.58 (0.53,** **10.69)**	**6.58 (1.52,** **11.66)**	**1.46 (-1.21, 4.19)**	2.95 (-0.88, 6.82)	**NRT+CRT+ SRT**	
-2 (-4.82, 0.83)	-1.08 (-3.92, 1.75)	0.56 (-1.9, 3.02)	0.27 (-1.95, 2.48)	**5.08 (1.94, 8.2)**	1.16 (-2.21, 4.57)	0.18 (-2.34, 2.71)	-0.04 (-3.16, 3.03)	**5.18 (1.2,** **9.19)**	**6.17 (2.19,** **10.14)**	**1.06 (-1.6,** **3.73)**	**2.55 (0.33,** **4.76)**	-0.4 (-3.59, 2.73)	**NFT+SFT+ NRT**

(The data in the table are the MD (95CrI) of the intervention in the row vs the intervention in the column. A larger MD denotes a higher VAS; 0 denotes no distinction between the two interventions. Bold denotes a statistically significant difference in pain.).

**Figure 5:**
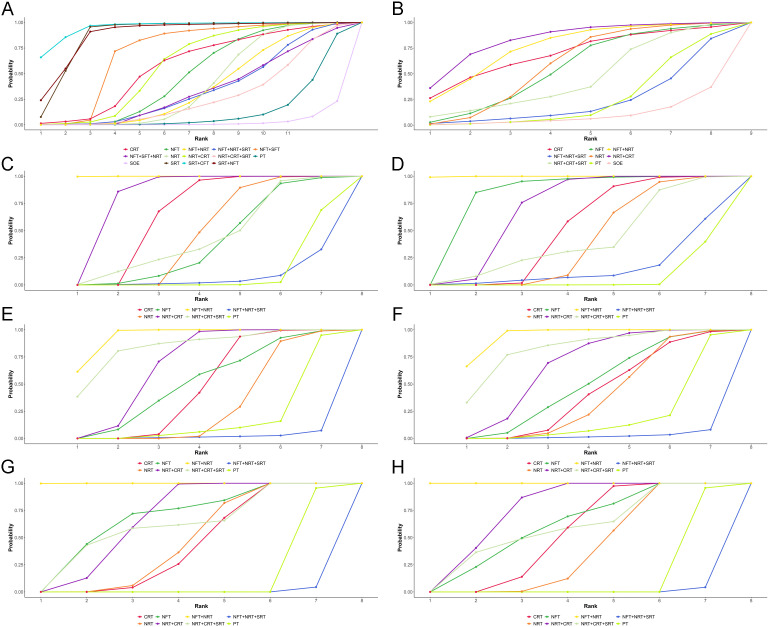
Cumulative ranking probability plots for each outcome. SUCRA values were used to describe the relative ranking probabilities of the competing interventions for each outcome. These rankings should be interpreted together with effect estimates, 95% CrIs, and certainty of evidence. [**(A)** VAS; **(B)** NDI; **(C)** Neck flexion ROM; **(D)** Neck extension ROM; **(E)** Neck left lateral flexion ROM; **(F)** Neck right lateral flexion ROM; **(G)** Neck left rotation ROM; **(H)** Neck right rotation ROM].

The SUCRA probability ranking for VAS was as follows: SRT+CFT (95.53%) > SRT+NFT (88.70%) > SRT (88.38%). SRT+CFT had the highest SUCRA value for VAS (**Figure 5A**). The CINeMA certainty-of-evidence assessment for VAS comparisons is presented in **Supplementary Table 8**.

##### NDI

A total of 15 studies reported NDI, including 745 patients. The NMA implied no significant difference in NDI between SOE and various training interventions in patients with NSNP. Detailed pairwise comparisons are presented in **Table 4**. The forest plot is depicted in **Figure 4B**.

**Table 4:** League table for NDI.

**SOE**								
-7.53 (-27.01, 12.59)	**PT**							
-19.86 (-49.19, 9.77)	-12.37 (-34.38, 9.51)	**NFT**						
-20.57 (-48.13, 7.69)	-13.05 (-32.69, 6.69)	-0.7 (-10.19, 9.27)	**NRT**					
-24.33 (-60.51, 12.29)	-16.76 (-47.23, 13.74)	-4.39 (-29.76, 20.84)	-3.67 (-27.09, 19.52)	**CRT**				
-24.66 (-54.39, 5.62)	-17.14 (-39.88, 5.45)	-4.77 (-15.68, 6.25)	-4.07 (-15.33, 6.91)	-0.39 (-26.19, 25.57)	**NFT+NRT**			
-5.67 (-22.77, 11.41)	1.87 (-15.71, 18.99)	14.21 (-13.82, 42.11)	14.91 (-11.67, 40.89)	18.62 (-16.81, 53.56)	18.97 (-9.72, 47.41)	**NFT+NRT+SRT**		
-27.43 (-58.28, 3.9)	-19.89 (-43.78, 4.02)	-7.52 (-24.19, 9.24)	-6.83 (-20.61, 6.56)	-3.15 (-22.04, 15.86)	-2.74 (-20.35, 14.79)	-21.75 (-51.08, 8.02)	**NRT+CRT**	
-13.57 (-29.95, 3.45)	-6.01 (-23.12, 11.1)	6.33 (-21.26, 34.31)	7.04 (-19.11, 33.17)	10.75 (-24.12, 45.81)	11.1 (-17.09, 39.69)	-7.87 (-27.21, 12.1)	13.88 (-15.49, 43.39)	**NRT+CRT+SRT**

(The data in the table are the MD (95CrI) of the intervention in the row vs the intervention in the column. A larger MD denotes a higher NDI; 0 denotes no distinction between the two interventions. Bold denotes a statistically significant difference in NDI.).

The SUCRA probability ranking for NDI was as follows: NRT+CRT (83.74%) > NFT+NRT (76.42%) > CRT (69.60%). NRT+CRT had the highest SUCRA value for NDI (**Figure 5B**). The CINeMA certainty-of-evidence assessment for NDI comparisons is presented in **Supplementary Table 9**.

#### Secondary outcomes

##### Neck flexion ROM

Eleven studies reported neck flexion ROM, including 555 patients. The NMA suggested that, compared to PT, NRT, CRT, NFT+NRT, SRT+NFT, NRT+CRT, and NRT+CRT+SRT significantly improved neck flexion ROM in patients with NSNP. Detailed pairwise comparisons are presented in **Table 5** (PT vs NRT: MD = 5.56, 95%CrI: 1.65,9.48; PT vs CRT: MD = 6.43, 95%CrI: 2.48,10.38; PT vs NFT+NRT: MD = 12.81,95%CrI: 8.05, 17.54; PT vs NRT+CRT: MD = 7.67,95%CrI: 3.73, 11.61; PT vs NRT+CRT+SRT: MD = 4.49, 95%CrI: 0.83, 8.19). The forest plot is illustrated in **Figure 4C**.

**Table 5:** League table for neck flexion ROM.

**PT**							
4.61 (-0.17, 9.4)	**NFT**						
**5.56 (1.65, 9.48)**	0.95 (-1.77, 3.67)	**NRT**					
**6.43 (2.48, 10.38)**	1.82 (-0.97, 4.61)	**0.87 (0.28, 1.46)**	**CRT**				
**12.81 (8.05, 17.54)**	**8.19 (6.81, 9.56)**	**7.24 (4.58, 9.88)**	**6.38 (3.65, 9.09)**	**NFT+NRT**			
-1.38 (-7.23, 4.51)	-5.99 (-13.53, 1.61)	-6.93 (-14, 0.13)	**-7.8 (-14.87, -0.71)**	**-14.17 (-21.69, -6.61)**	**NFT+NRT+SRT**		
**7.67 (3.73, 11.61)**	**3.06 (0.3, 5.83)**	**2.11 (1.63, 2.59)**	**1.24 (0.9, 1.58)**	**-5.13 (-7.82, -2.44)**	**9.03 (1.95, 16.12)**	**NRT+CRT**	
**4.49 (0.83, 8.19)**	-0.12 (-6.17, 5.92)	-1.07 (-6.45, 4.33)	-1.94 (-7.36, 3.48)	**-8.31 (-14.3, -2.31)**	5.88 (-1.08, 12.8)	-3.18 (-8.58, 2.23)	**NRT+CRT+SRT**

(The data in the table are the MD (95CrI) of the intervention in the row vs the intervention in the column. A larger MD denotes a higher neck flexion ROM; 0 denotes no distinction between the two interventions. Bold denotes a statistically significant distinction in neck flexion ROM.).

The SUCRA probability ranking for neck flexion ROM was as follows: NFT+NRT (99.95%) > NRT+CRT (83.63%) > CRT (66.27%). NFT+NRT had the highest SUCRA value for neck flexion ROM (**Figure 5C**). The CINeMA certainty-of-evidence assessment for neck flexion ROM comparisons is presented in **Supplementary Table 10**.

##### Neck extension ROM

Ten studies reported neck extension ROM, including 515 patients. The NMA implied that, relative to PT, NFT, NRT, CRT, NFT+NRT, NRT+CRT, and NRT+CRT+SRT significantly improved neck extension ROM in patients with NSNP. Detailed pairwise comparisons are presented in **Table 6** (PT vs NFT: MD = 8.71, 95%CrI: 3.83, 13.58; PT vs NRT: MD = 5.57, 95%CrI: 1.67, 9.48; PT vs CRT: MD = 5.82, 95%CrI: 1.89, 9.76; PT vs NFT+NRT: MD = 12.22, 95%CrI: 7.36, 17.06; PT vs NRT+CRT: MD = 6.51, 95%CrI: 2.6, 10.44; PT vs NRT+CRT+SRT: MD = 4.51, 95%CrI: 0.81, 8.2). The forest plot is illustrated in **Figure 4D**.

**Table 6:** League table for neck extension ROM.

**PT**							
**8.71 (3.83, 13.58)**	**NFT**						
**5.57 (1.67, 9.48)**	**-3.14 (-6.04, -0.21)**	**NRT**					
**5.82 (1.89, 9.76)**	-2.89 (-5.83, 0.08)	0.25 (-0.18, 0.68)	**CRT**				
**12.22 (7.36, 17.06)**	**3.51 (2.27, 4.76)**	**6.65 (3.74, 9.52)**	**6.4 (3.47, 9.31)**	**NFT+NRT**			
0.81 (-5, 6.62)	**-7.91 (-15.44, -0.24)**	-4.77 (-11.78, 2.27)	-5.02 (-12.03, 2.03)	**-11.42 (-18.96, -3.77)**	**NFT+NRT+SRT**		
**6.51 (2.6, 10.44)**	-2.2 (-5.12, 0.75)	**0.94 (0.64, 1.24)**	**0.69 (0.38, 1)**	**-5.71 (-8.6, -2.79)**	5.71 (-1.34, 12.72)	**NRT+CRT**	
**4.51 (0.81, 8.2)**	-4.21 (-10.34, 1.93)	-1.07 (-6.45, 4.3)	-1.32 (-6.73, 4.07)	**-7.72 (-13.82, -1.61)**	3.69 (-3.19, 10.55)	-2.01 (-7.4, 3.37)	**NRT+CRT+SRT**

(The data in the table are the MD (95CrI) of the intervention in the row vs the intervention in the column. A larger MD denotes a higher neck extension ROM; 0 denotes no distinction between the two interventions. Bold denotes a statistically significant distinction in neck extension ROM.).

The SUCRA probability ranking for neck extension ROM was as follows: NFT+NRT (99.87%) > NFT (82.43%) > NRT+CRT (68.33%). NFT+NRT had the highest SUCRA value for neck extension ROM (**Figure 5D**). The CINeMA certainty-of-evidence assessment for neck extension ROM comparisons is presented in **Supplementary Table 11**.

##### Neck left lateral flexion ROM

Nine studies reported neck left lateral flexion ROM, including 489 patients. The NMA showed that, compared to PT, NFT+NRT and NRT+CRT+SRT significantly improved neck left lateral flexion ROM in patients with NSNP. Detailed pairwise comparisons are presented in **Table 7**(PT vs NFT+NRT: MD = 5.57, 95%CrI: 1.95, 9.22; PT vs NRT+CRT+SRT: MD = 4.87, 95%CrI: 1.89, 7.85). The forest plot is illustrated in **Figure 4E**.

**Table 7:** League table for neck left lateral flexion ROM.

**PT**							
2.3 (-1.27, 5.9)	**NFT**						
1.72 (-1.26, 4.72)	-0.59 (-2.56, 1.39)	**NRT**					
2.06 (-0.92, 5.08)	-0.25 (-2.23, 1.74)	**0.34 (0.15, 0.53)**	**CRT**				
**5.57 (1.95, 9.22)**	**3.26 (1.97, 4.55)**	**3.85 (1.77, 5.93)**	**3.51 (1.43, 5.59)**	**NFT+NRT**			
-3.22 (-7.31, 0.83)	**-5.52 (-10.94, -0.14)**	-4.93 (-9.99, 0.06)	**-5.28 (-10.32, -0.28)**	**-8.78 (-14.26, -3.36)**	**NFT+NRT+SRT**		
2.78 (-0.2, 5.79)	0.47 (-1.51, 2.46)	**1.06 (0.94, 1.18)**	**0.72 (0.57, 0.87)**	**-2.79 (-4.87, -0.71)**	**6 (1.01, 11.04)**	**NRT+CRT**	
**4.87 (1.89, 7.85)**	2.56 (-2.11, 7.2)	3.15 (-1.09, 7.34)	2.81 (-1.43, 7.01)	-0.7 (-5.42, 3.99)	**8.09 (3.04, 13.14)**	2.08 (-2.15, 6.28)	**NRT+CRT+SRT**

(The data in the table are the MD (95CrI) of the intervention in the row vs the intervention in the column. A larger MD denotes a higher neck left lateral flexion ROM; 0 denotes no distinction between the two interventions. Bold denotes a statistically significant distinction in neck left lateral flexion ROM.).

The SUCRA probability ranking for neck left lateral flexion ROM was as follows: NFT+NRT (94.40%) > NRT+CRT+SRT (84.42%) > NRT+CRT (68.67%). NFT+NRT had the highest SUCRA value for neck left lateral flexion ROM (**Figure 5E**). The CINeMA certainty-of-evidence assessment for neck left lateral flexion ROM comparisons is presented in **Supplementary Table 12**.

##### Neck right lateral flexion ROM

Nine studies reported neck right lateral flexion ROM, involving 489 patients. The NMA suggested that, compared to PT, NFT+NRT and NRT+CRT+SRT significantly increased neck right lateral flexion ROM in patients with NSNP. Detailed pairwise comparisons are presented in **Table 8** (PT vs NFT+NRT: MD = 5.89, 95% CrI: 2.46, 9.3; PT vs NRT+CRT+SRT: MD = 4.87, 95% CrI: 1.91, 7.85). The forest plot is depicted in **Figure 4F**.

**Table 8:** League table for neck right lateral flexion ROM.

**PT**							
2.16 (-1.33, 5.61)	**NFT**						
1.73 (-1.28, 4.74)	-0.43 (-2.17, 1.3)	**NRT**					
1.97 (-1.71, 5.65)	-0.18 (-2.95, 2.54)	0.26 (-1.91, 2.33)	**CRT**				
**5.89 (2.46, 9.3)**	**3.73 (2.4, 5.07)**	**4.15 (2.56, 5.77)**	**3.91 (1.27, 6.6)**	**NFT+NRT**			
-3.23 (-7.3, 0.86)	**-5.39 (-10.71, -0.02)**	-4.95 (-9.98, 0.11)	-5.19 (-10.68, 0.27)	**-9.12 (-14.43, -3.79)**	**NFT+NRT+SRT**		
2.92 (-0.74, 6.57)	0.78 (-1.98, 3.45)	1.21 (-0.92, 3.25)	**0.95 (0.56, 1.34)**	**-2.96 (-5.62, -0.36)**	**6.15 (0.69, 11.62)**	**NRT+CRT**	
**4.87 (1.91, 7.85)**	2.72 (-1.83, 7.29)	3.15 (-1.06, 7.37)	2.91 (-1.81, 7.61)	-1.01 (-5.52, 3.52)	**8.11 (3.09, 13.16)**	1.96 (-2.74, 6.64)	**NRT+CRT+SRT**

(The data in the table are the MD (95CrI) of the intervention in the row vs the intervention in the column. A larger MD denotes a higher neck right lateral flexion ROM; 0 denotes no distinction between the two interventions. Bold denotes a statistically significant distinction in neck right lateral flexion ROM.).

The SUCRA probability ranking for neck right lateral flexion ROM was as follows: NFT+NRT (95.04%) > NRT+CRT+SRT (83.10%) > NRT+CRT (67.51%). NFT+NRT had the highest SUCRA value for neck right lateral flexion ROM (**Figure 5F**). The CINeMA certainty-of-evidence assessment for neck right lateral flexion ROM comparisons is presented in **Supplementary Table 13**.

##### Neck left rotation ROM

Nine studies reported neck left rotation ROM, including 489 patients. The NMA suggested that, relative to PT, NFT, NRT, CRT, NFT+NRT, NRT+CRT, and NRT+CRT+SRT significantly increased neck left rotation ROM in patients with NSNP. Detailed pairwise comparisons are presented in **Table 9** (PT vs NFT: MD = 8.44,95%CrI: 3.57, 13.29; PT vs NRT: MD = 7.3, 95%CrI: 3.51, 11.08; PT vs CRT: MD = 7.24, 95%CrI: 3.42, 11.05; PT vs NFT+NRT: MD = 17.49, 95%CrI: 12.57, 22.38; PT vs NRT+CRT: MD = 7.64, 95%CrI: 3.83, 11.44; PT vs NRT+CRT+SRT: MD = 8.17, 95%CrI: 4.09, 12.25). The forest plot is illustrated in **Figure 4G**.

**Table 9:** League table for neck left rotation ROM.

**PT**							
**8.44 (3.57, 13.29)**	**NFT**						
**7.3 (3.51, 11.08)**	-1.14 (-4.16, 1.92)	**NRT**					
**7.24 (3.42, 11.05)**	-1.2 (-4.24, 1.88)	-0.06 (-0.46, 0.34)	**CRT**				
**17.49 (12.57, 22.38)**	**9.06 (7.77, 10.33)**	**10.19 (7.08, 13.28)**	**10.25 (7.12, 13.35)**	**NFT+NRT**			
-4.11 (-8.86, 0.63)	**-12.55 (-19.33, -5.75)**	**-11.41 (-17.52, -5.34)**	**-11.35 (-17.46, -5.27)**	**-21.6 (-28.42, -14.76)**	**NFT+NRT+SRT**		
**7.64 (3.83, 11.44)**	-0.8 (-3.83, 2.27)	**0.34 (0.04, 0.64)**	**0.4 (0.13, 0.67)**	**-9.85 (-12.94, -6.73)**	**11.75 (5.68, 17.86)**	**NRT+CRT**	
**8.17 (4.09, 12.25)**	-0.26 (-6.63, 6.09)	0.87 (-4.71, 6.44)	0.93 (-4.65, 6.52)	**-9.31 (-15.7, -2.94)**	**12.29 (6.05, 18.56)**	0.53 (-5.06, 6.11)	**NRT+CRT+SRT**

(The data in the table are the MD (95CrI) of the intervention in the row vs the intervention in the column. A larger MD denotes a higher neck left rotation ROM; 0 denotes no distinction between the two interventions. Bold denotes a statistically significant distinction in neck left rotation ROM.).

The SUCRA probability ranking for neck left rotation ROM was as follows: NFT+NRT (99.97%) > NFT (68.18%) > NRT+CRT (67.37%). NFT+NRT had the highest SUCRA value for neck left rotation ROM (**Figure 5G**). The CINeMA certainty-of-evidence assessment for neck left rotation ROM comparisons is presented in **Supplementary Table 14**.

##### Neck right rotation ROM

Nine studies reported neck right rotation ROM, including 489 patients. The NMA indicated that, compared to PT, NFT, NRT, CRT, NFT+NRT, NRT+CRT, and NRT+CRT+SRT significantly increased neck right rotation ROM in patients with NSNP. Detailed pairwise comparisons are presented in **Table 10** (PT vs NFT: MD = 8.23,95%CrI: 3.54, 12.98; PT vs NRT: MD = 7.31, 95%CrI: 3.51, 11.09; PT vs CRT: MD = 7.62, 95%CrI: 3.81, 11.43; PT vs NFT+NRT: MD = 18.97, 95%CrI: 14.24, 23.72; PT vs NRT+CRT: MD = 8.89, 95%CrI: 5.09, 12.69; PT vs NRT+CRT+SRT: MD = 8.18,95%CrI: 4.1,12.28). The forest plot is illustrated in **Figure 4H**.

**Table 10:** League table for neck right rotation ROM.

**PT**							
**8.23 (3.54, 12.98)**	**NFT**						
**7.31 (3.51, 11.09)**	-0.93 (-3.76, 1.87)	**NRT**					
**7.62 (3.81, 11.43)**	-0.61 (-3.47, 2.23)	0.32 (-0.07, 0.71)	**CRT**				
**18.97 (14.24, 23.72)**	**10.73 (9.36, 12.1)**	**11.67 (8.81, 14.51)**	**11.34 (8.46, 14.22)**	**NFT+NRT**			
-4.13 (-8.86, 0.59)	**-12.37 (-19.05, -5.69)**	**-11.44 (-17.49, -5.4)**	**-11.76 (-17.81, -5.7)**	**-23.11 (-29.78, -16.43)**	**NFT+NRT+SRT**		
**8.89 (5.09, 12.69)**	0.66 (-2.18, 3.48)	**1.59 (1.33, 1.85)**	**1.27 (0.97, 1.57)**	**-10.08 (-12.94, -7.21)**	**13.03 (6.98, 19.08)**	**NRT+CRT**	
**8.18 (4.1, 12.28)**	-0.05 (-6.3, 6.19)	0.88 (-4.67, 6.49)	0.56 (-5.01, 6.18)	**-10.79 (-17.03, -4.53)**	**12.31 (6.1, 18.57)**	-0.71 (-6.27, 4.91)	**NRT+CRT+SRT**

(The data in the table are the MD (95CrI) of the intervention in the row vs the intervention in the column. A larger MD denotes a higher neck right rotation ROM; 0 denotes no distinction between the two interventions. Bold denotes a statistically significant distinction in neck right rotation ROM.).

The SUCRA probability ranking for neck right rotation ROM was as follows: NFT+NRT (99.995%) > NRT+CRT (75.34%) > NFT (60.49%). NFT+NRT had the highest SUCRA value for neck right rotation ROM (**Figure 5H**). The CINeMA certainty-of-evidence assessment for neck right rotation ROM comparisons is presented in **Supplementary Table 15**.

### Publication bias

**Figure 6:**
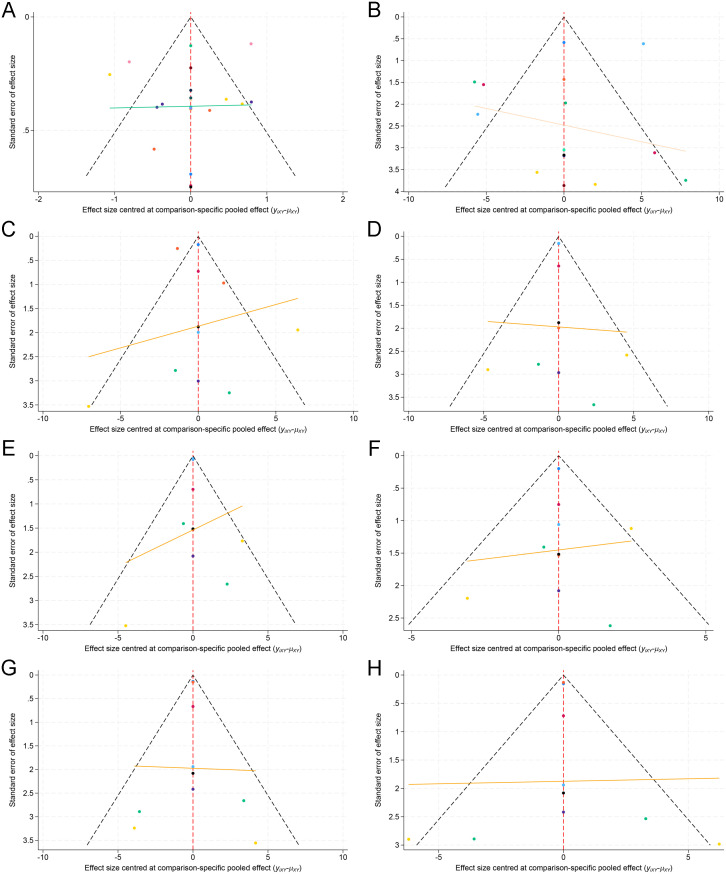
Comparative corrected funnel plot of outcome indicators. The figure is used to assess potential small sample effects and publication bias in network meta-analysis. A descriptive analysis of the symmetry of the funnel plot was conducted. However, due to the limited number of studies and the sparse treatment nodes, the ability to detect small sample effects or publication bias is limited. [**(A)** VAS; **(B)** NDI; **(C)** Neck flexion ROM; **(D)** Neck extension ROM; **(E)** Neck left lateral flexion ROM; **(F)** Neck right lateral flexion ROM; **(G)** Neck left rotation ROM; **(H)** Neck right rotation ROM].

Publication bias was tested by plotting corrected-comparison funnel plots. The adjusted funnel plots were generally symmetrical. However, due to the limited number of studies and their distribution across multiple treatment nodes, our ability to detect small sample effects or publication bias was limited. Therefore, the possibility of publication bias could not be ruled out (**Figure 6**).

## Discussion

This NMA compared the efficacy of different stability training strategies for NSNP. The overall results showed that the effects of stability exercise-based interventions on different outcomes were inconsistent. In general, NRT, SRT, CRT, NFT, and their combinations showed more positive effects in relieving pain and improving ROM of joints of the cervical vertebra, but did not show a clear advantage in alleviating functional impairment. These results are generally consistent with the conclusions of existing clinical practice guidelines and high-quality reviews, that is, active exercise therapy should be used as an important conservative treatment for chronic neck pain or NSNP, but the relative merits of different training components remain unclear (Blanpied et al., 2017; de Zoete, 2023; Mueller et al., 2023). Furthermore, this study indicated that the clinical effects of different training components and their combinations of stability exercise were not identical. Combination strategies potentially were more beneficial in relieving pain and improving ROM. However, they failed to significantly change NDI, suggesting that symptom relief and functional recovery may not be entirely consistent (Mueller et al., 2023).

Among the various outcomes, VAS was more sensitive to the intervention effect (Hawker et al., 2011; MacDowall et al., 2018). This study showed that stability exercise-based interventions could reduce pain to varying degrees. Local resistance training and its combination strategies showed the most significant effects. These results are consonant with the overall conclusions of previous guidelines and reviews, that is, active training incorporating reinforcement, motor control, and stability components is an important treatment for chronic NSNP, and generally demonstrates good responsiveness in terms of pain relief (Cohen, 2015; Blanpied et al., 2017; Mueller et al., 2023). Its underlying mechanism may be related to improvements in local neuromuscular function. Resistance training and control training may help optimize the recruitment patterns of the deep neck flexors and scapular stabilizers, may improve load distribution in the neck and shoulder region, and possibly relieve pain by enhancing exercise confidence and self-efficacy; this explanation is broadly consistent with the findings of existing studies on deep cervical flexor training and scapular training (Tsiringakis et al., 2020; Iqbal et al., 2021; Bharti et al., 2024; Chen et al., 2024). Therefore, stability exercise-based combination strategies may be significantly beneficial in pain relief because it simultaneously acts on multiple aspects such as local control, muscle strength, and load regulation.

Unlike pain outcomes, NDI reflects a more comprehensive state of functional limitation (Vernon and Mior, 1991; Vernon, 2008). This study showed that although intervention strategies based on stability exercise exhibit some advantages in reducing pain and enhancing ROM, they did not show consistently significant effects on improving NDI. These results are largely consistent with previous reviews (Cho et al., 2023; Mueller et al., 2023), that is, exercise therapy is generally effective in improving function in chronic NSNP. However, the differences between different training types are usually small, and functional improvements are often less sensitive than pain relief (Cho et al., 2023; Mueller et al., 2023). This result suggests that functional recovery may lag behind symptom improvement, possibly because functional impairment is not only affected by local pain and limited mobility, but also by factors such as psychological state, behavioral patterns, self-efficacy, and daily mechanical workload (Cho et al., 2023; de Zoete, 2023; Mueller et al., 2023). Therefore, no synchronous significant changes in NDI observed in this study more likely reflect the complexity and multidimensionality of functional recovery in NSNP patients.

ROM is an important objective indicator for assessing localized movement limitation and functional recovery (Howell, 2011; Snodgrass et al., 2014). This study found that intervention strategies based on stability exercise, especially NFT+NRT, are more beneficial in improving ROM of cervical flexion, extension, lateral flexion, and rotation. This result is consistent with the overall conclusions of existing guidelines and related studies, that is, neck mobilization training, strengthening training, and neuromuscular control training all help improve motor function of the cervical spine, and are of particular clinical significance in individuals with marked localized mobility restrictions (Blanpied et al., 2017; Tsiringakis et al., 2020; Bharti et al., 2024; Chaiyawijit and Kanlayanaphotporn, 2024). The underlying mechanism may lie in the fact that flexibility training helps relieve the tension of soft tissue and protective muscle spasms, while resistance training helps enhance the active control of neck muscles and joint stability. The combined effect of both is more conducive to restoring coordination and ROM in multiplanar movements of the cervical spine. Therefore, combined training may have a greater advantage in improving ROM.

The findings of this study have certain clinical implications. First, not all components of a stability exercise-based treatment strategy have the same effect. Different components should be selected based on different treatment goals. For patients whose primary treatment goals are to relieve pain and restore ROM, neck local control training, resistance training, and their combinations may be considered. Second, since improvements in NDI are not significant, we cannot solely use pain relief or ROM recovery as treatment endpoints in clinical practice. Instead, a more comprehensive rehabilitation plan should be developed based on the patient’s functional limitations, psychological state, self-management ability, and daily workload. In other words, stability exercise may be an important component of rehabilitation plans for NSNP, but to achieve more comprehensive and sustained functional benefits, it still needs to be combined with other strategies such as education, behavioral management, and long-term family training.This is also in line with current trends in rehabilitation management, which emphasize individualization and a multidimensional approach (Blanpied et al., 2017; de Zoete, 2023).

This study has several strengths. First, we conducted a relatively extensive literature search, systematically integrating current evidence from RCTs on NSNP interventions based on stability training. Second, NMA was used to simultaneously compare different training components and their combinations, which is more effective than traditional pairwise meta-analysis in revealing the relative efficacy between different strategies. Furthermore, this study included pain, functional impairment, and multi-directional ROM of the cervical spine, evaluating the intervention effect from multiple dimensions. Nonetheless, this NMA has several limitations. First, the overall number of included studies was limited, and some treatment nodes and outcomes were informed by only a small number of studies, which reduced the precision of several comparisons. Second, the eligible studies were geographically concentrated, which may limit the generalizability of the findings. Third, direct head-to-head comparisons between several intervention nodes were scarce, and some network estimates relied mainly on indirect evidence, which may weaken the robustness of the results. Fourth, despite the use of prespecified operational definitions, variability in exercise intensity, supervision, frequency, and progression across studies may have contributed to clinical heterogeneity. Fifth, although key effect modifiers were descriptively examined, potential violations of the transitivity assumption cannot be fully excluded because symptom duration, baseline severity, and treatment duration varied across trials. Sixth, the certainty of evidence was generally low or very low across key comparisons, with only a few moderate-certainty estimates. This should be considered when interpreting the rankings and the apparent superiority of some interventions. Therefore, the findings, especially those for functional outcomes and some ROM comparisons, should be interpreted with caution.

Furthermore, in several comparisons, the number of studies included at each treatment node was relatively small. The evidence for head-to-head comparisons remained limited, treatment durations and participant characteristics varied across different trials. Potential violations of the transitivity hypothesis could not be completely ruled out. In addition, most of the included studies assessed short-term outcomes and did not formally assess evidence certainty. This factor should be considered when interpreting the results.

Future NMA needs to include more well-designed RCTs with sufficient sample sizes to improve the stability of comparisons across interventions. Simultaneously, original studies should report key elements such as training frequency, session duration, total number of sessions, intensity, supervision methods, and adherence in a more standardized manner to support subsequent subgroup analyses, meta-regression analyses, and dose-response analyses. Furthermore, future research should further clarify the independent effects and combined effects of different stability exercise components, standardize the diagnostic criteria, control settings, follow-up time points, and outcome measurement tools for NSNP, and simultaneously incorporate indicators such as psychological factors, behavioral characteristics, and long-term functional recovery, thereby providing a more solid evidence base for developing more precise and implementable clinical exercise prescriptions.

The mechanism explanations above should be interpreted with caution, as they are intended to provide explanations for the research results rather than directly reflect the validated mechanisms included in the trials.

## Conclusion

This NMA suggests that NRT, SRT, CRT, NFT, and their combinations may be associated with greater pain relief and ROM improvement in patients with NSNP, whereas no clear advantage was found for functional recovery. However, given the generally low or very low certainty of evidence and the sparse evidence in several treatment nodes, these findings should be interpreted cautiously.

## Data Availability

The original contributions presented in the study are included in the article/**Supplementary Material**. Further inquiries can be directed to the corresponding author.
