# Supplementary material for: Efficacy of stability exercise-based treatment strategies for non-specific neck pain: a network meta-analysis
Source: Front Physiol. 2026 Jul 23;17:1878997. doi: 10.3389/fphys.2026.1878997 (PMC13442010; doi:10.3389/fphys.2026.1878997)
Supplement: Supplementary file 1 [file DataSheet1.docx]

**Supplementary Materials**

**Supplementary Table S1** Operational definitions and coding rules for intervention nodes.

| **Node** | **Primary target region** | **Dominant training intent** | **Operational definition** |
| --- | --- | --- | --- |
| NFT | Neck | Flexibility | Exercise primarily intended to improve cervical flexibility, movement amplitude, or soft-tissue extensibility |
| NRT | Neck | Resistance | Exercise primarily intended to improve neck muscle strength or endurance through active force generation against resistance |
| SFT | Shoulder girdle | Flexibility | Exercise primarily intended to improve shoulder girdle flexibility or scapulothoracic mobility |
| SRT | Shoulder girdle | Resistance | Exercise primarily intended to improve shoulder girdle or scapular muscle strength/endurance |
| CFT | Chest | Flexibility | Exercise primarily intended to improve chest/anterior thoracic soft-tissue extensibility or thoracic opening |
| CRT | Core | Resistance | Exercise primarily intended to improve trunk/core strength or endurance |
| Combination nodes | Multiple | Mixed | Interventions including two or more predefined components delivered within the same treatment program |

**Supplementary Table S2** Literature search strategy.

**(Pubmed)**

| Search number | Query | Results |
| --- | --- | --- |
| 1 | Neck Pain[MeSH Terms] | 9,577 |
| 2 | "Anterior Cervical Pains"[Title/Abstract] OR "Anterior Neck Pains"[Title/Abstract] OR "Anterior Neck Pain"[Title/Abstract] OR "Cervical Pain"[Title/Abstract] OR "Cervical Pains"[Title/Abstract] OR "Cervicalgia"[Title/Abstract] OR "Cervicalgias"[Title/Abstract] OR "Cervicodynia"[Title/Abstract] OR "Cervicodynias"[Title/Abstract] OR "Neck Ache"[Title/Abstract] OR "Neck Aches"[Title/Abstract] OR "neck pain"[Title/Abstract] OR "Neck Pains"[Title/Abstract] OR "Neckache"[Title/Abstract] OR "Neckaches"[Title/Abstract] OR "pain, neck"[Title/Abstract] OR "Posterior Cervical Pain"[Title/Abstract] OR "Posterior Cervical Pains"[Title/Abstract] OR "Posterior Neck Pains"[Title/Abstract] OR "Posterior Neck Pain"[Title/Abstract] | 16,719 |
| 3 | "Nonspecific"[Title/Abstract] OR "non specific"[Title/Abstract] OR "non-specific"[Title/Abstract] | 157,527 |
| 4 | ((Resistance Training[MeSH Terms]) OR (Motion Therapy, Continuous Passive[MeSH Terms])) OR (Muscle Stretching Exercises[MeSH Terms]) | 17,688 |
| 5 | "Active Stretching"[Title/Abstract] OR "Ballistic Stretching"[Title/Abstract] OR "Continuous Passive Motion Therapy"[Title/Abstract] OR "Continuous Passive Movement Therapy"[Title/Abstract] OR "core stabilisation exercise"[Title/Abstract] OR "core stabilisation training"[Title/Abstract] OR "core stability exercise"[Title/Abstract] OR "core stability training"[Title/Abstract] OR "core stabilization exercise"[Title/Abstract] OR "core stabilization training"[Title/Abstract] OR "CPM Therapies"[Title/Abstract] OR "CPM Therapy"[Title/Abstract] OR "Dynamic Stretching"[Title/Abstract] OR "Isometric Stretching"[Title/Abstract] OR "motion therapy"[Title/Abstract] OR "movement based therapy"[Title/Abstract] OR "movement therapy"[Title/Abstract] OR "Muscle Stretching Exercise"[Title/Abstract] OR "Muscle Stretching Exercises"[Title/Abstract] OR "Passive Stretching"[Title/Abstract] OR "PNF Stretching"[Title/Abstract] OR "PNF Stretching Exercise"[Title/Abstract] OR "PNF Stretching Exercises"[Title/Abstract] OR "PNF Stretchings"[Title/Abstract] OR "Proprioceptive Neuromuscular Facilitation"[Title/Abstract] OR "Proprioceptive Neuromuscular Facilitation (PNF) Stretching"[Title/Abstract] OR "Proprioceptive Neuromuscular Facilitations"[Title/Abstract] OR "Relaxed Stretching"[Title/Abstract] OR "resistance exercise"[Title/Abstract] OR "resistance exercise training"[Title/Abstract] OR "Resistance Training"[Title/Abstract] OR "resistance type exercise"[Title/Abstract] OR "resistance type training"[Title/Abstract] OR "stabilisation exercise"[Title/Abstract] OR "stabilisation training"[Title/Abstract] OR "Stability Training"[Title/Abstract] OR "stabilization exercise"[Title/Abstract] OR "stabilization training"[Title/Abstract] OR "Static Active Stretching"[Title/Abstract] OR "Static Passive Stretching"[Title/Abstract] OR "Static Stretching"[Title/Abstract] OR "Strength Training"[Title/Abstract] OR "strength type exercise"[Title/Abstract] OR "strength type training"[Title/Abstract] OR "stretching exercise"[Title/Abstract] OR "stretching exercises"[Title/Abstract] OR "Weight Bearing Exercise Program"[Title/Abstract] OR "Weight Bearing Exercise Programs"[Title/Abstract] OR "Weight Bearing Strengthening Program"[Title/Abstract] OR "Weight Bearing Strengthening Programs"[Title/Abstract] OR "Weight Lifting Exercise Program"[Title/Abstract] OR "Weight Lifting Exercise Programs"[Title/Abstract] OR "Weight Lifting Strengthening Program"[Title/Abstract] OR "Weight Lifting Strengthening Programs"[Title/Abstract] | 32,991 |
| 6 | (#1 OR #2) AND #3 AND (#4 OR #5) | 60 |

**(Embase)**

| Search number | Query | Results |
| --- | --- | --- |
| 1 | 'neck pain'/exp | 38,163 |
| 2 | 'anterior cervical pains':ab,ti,kw OR 'anterior neck pains':ab,ti,kw OR 'anterior neck pain':ab,ti,kw OR 'cervical pain':ab,ti,kw OR 'cervical pains':ab,ti,kw OR 'cervicalgia':ab,ti,kw OR 'cervicalgias':ab,ti,kw OR 'cervicodynia':ab,ti,kw OR 'cervicodynias':ab,ti,kw OR 'neck ache':ab,ti,kw OR 'neck aches':ab,ti,kw OR 'neck pain':ab,ti,kw OR 'neck pains':ab,ti,kw OR 'neckache':ab,ti,kw OR 'neckaches':ab,ti,kw OR 'pain, neck':ab,ti,kw OR 'posterior cervical pain':ab,ti,kw OR 'posterior cervical pains':ab,ti,kw OR 'posterior neck pains':ab,ti,kw OR 'posterior neck pain':ab,ti,kw | 25,420 |
| 3 | 'nonspecific':ab,ti,kw OR 'non specific':ab,ti,kw OR 'non-specific':ab,ti,kw | 214,128 |
| 4 | 'core stability training'/exp OR 'resistance training'/exp OR 'stretching exercise'/exp OR 'movement therapy'/exp | 49,652 |
| 5 | 'active stretching':ab,ti,kw OR 'ballistic stretching':ab,ti,kw OR 'continuous passive motion therapy':ab,ti,kw OR 'continuous passive movement therapy':ab,ti,kw OR 'core stabilisation exercise':ab,ti,kw OR 'core stabilisation training':ab,ti,kw OR 'core stability exercise':ab,ti,kw OR 'core stability training':ab,ti,kw OR 'core stabilization exercise':ab,ti,kw OR 'core stabilization training':ab,ti,kw OR 'cpm therapies':ab,ti,kw OR 'cpm therapy':ab,ti,kw OR 'dynamic stretching':ab,ti,kw OR 'isometric stretching':ab,ti,kw OR 'motion therapy':ab,ti,kw OR 'movement based therapy':ab,ti,kw OR 'movement therapy':ab,ti,kw OR 'muscle stretching exercise':ab,ti,kw OR 'muscle stretching exercises':ab,ti,kw OR 'passive stretching':ab,ti,kw OR 'pnf stretching':ab,ti,kw OR 'pnf stretching exercise':ab,ti,kw OR 'pnf stretching exercises':ab,ti,kw OR 'pnf stretchings':ab,ti,kw OR 'proprioceptive neuromuscular facilitation':ab,ti,kw OR 'proprioceptive neuromuscular facilitation (pnf) stretching':ab,ti,kw OR 'proprioceptive neuromuscular facilitations':ab,ti,kw OR 'relaxed stretching':ab,ti,kw OR 'resistance exercise':ab,ti,kw OR 'resistance exercise training':ab,ti,kw OR 'resistance training':ab,ti,kw OR 'resistance type exercise':ab,ti,kw OR 'resistance type training':ab,ti,kw OR 'stabilisation exercise':ab,ti,kw OR 'stabilisation training':ab,ti,kw OR 'stability training':ab,ti,kw OR 'stabilization exercise':ab,ti,kw OR 'stabilization training':ab,ti,kw OR 'static active stretching':ab,ti,kw OR 'static passive stretching':ab,ti,kw OR 'static stretching':ab,ti,kw OR 'strength training':ab,ti,kw OR 'strength type exercise':ab,ti,kw OR 'strength type training':ab,ti,kw OR 'stretching exercise':ab,ti,kw OR 'stretching exercises':ab,ti,kw OR 'weight bearing exercise program':ab,ti,kw OR 'weight bearing exercise programs':ab,ti,kw OR 'weight bearing strengthening program':ab,ti,kw OR 'weight bearing strengthening programs':ab,ti,kw OR 'weight lifting exercise program':ab,ti,kw OR 'weight lifting exercise programs':ab,ti,kw OR 'weight lifting strengthening program':ab,ti,kw OR 'weight lifting strengthening programs':ab,ti,kw | 46,494 |
| 6 | (#1 OR #2) AND #3 AND (#4 OR #5) | 132 |

**(Cochrane)**

| Search number | Query | Results |
| --- | --- | --- |
| 1 | MeSH descriptor: [Neck Pain] explode all trees | 2,325 |
| 2 | ('Anterior Cervical Pains' OR 'Anterior Neck Pains' OR 'Anterior Neck Pain' OR 'Cervical Pain' OR 'Cervical Pains' OR 'Cervicalgia' OR 'Cervicalgias' OR 'Cervicodynia' OR 'Cervicodynias' OR 'Neck Ache' OR 'Neck Aches' OR 'neck pain' OR 'Neck Pains' OR 'Neckache' OR 'Neckaches' OR 'pain, neck' OR 'Posterior Cervical Pain' OR 'Posterior Cervical Pains' OR 'Posterior Neck Pains' OR 'Posterior Neck Pain'):ab,ti,kw | 16,549 |
| 3 | 'Nonspecific' OR 'non specific' OR 'non-specific' | 43,820 |
| 4 | MeSH descriptor: [Resistance Training] explode all trees | 6,264 |
| 5 | MeSH descriptor: [Motion Therapy, Continuous Passive] explode all trees | 160 |
| 6 | MeSH descriptor: [Muscle Stretching Exercises] explode all trees | 951 |
| 7 | 'Active Stretching' OR 'Ballistic Stretching' OR 'Continuous Passive Motion Therapy' OR 'Continuous Passive Movement Therapy' OR 'core stabilisation exercise' OR 'core stabilisation training' OR 'core stability exercise' OR 'core stability training' OR 'core stabilization exercise' OR 'core stabilization training' OR 'CPM Therapies' OR 'CPM Therapy' OR 'Dynamic Stretching' OR 'Isometric Stretching' OR 'motion therapy' OR 'movement based therapy' OR 'movement therapy' OR 'Muscle Stretching Exercise' OR 'Muscle Stretching Exercises' OR 'Passive Stretching' OR 'PNF Stretching' OR 'PNF Stretching Exercise' OR 'PNF Stretching Exercises' OR 'PNF Stretchings' OR 'Proprioceptive Neuromuscular Facilitation' OR 'Proprioceptive Neuromuscular Facilitation (PNF) Stretching' OR 'Proprioceptive Neuromuscular Facilitations' OR 'Relaxed Stretching' OR 'resistance exercise' OR 'resistance exercise training' OR 'Resistance Training' OR 'resistance type exercise' OR 'resistance type training' OR 'stabilisation exercise' OR 'stabilisation training' OR 'Stability Training' OR 'stabilization exercise' OR 'stabilization training' OR 'Static Active Stretching' OR 'Static Passive Stretching' OR 'Static Stretching' OR 'Strength Training' OR 'strength type exercise' OR 'strength type training' OR 'stretching exercise' OR 'stretching exercises' OR 'Weight Bearing Exercise Program' OR 'Weight Bearing Exercise Programs' OR 'Weight Bearing Strengthening Program' OR 'Weight Bearing Strengthening Programs' OR 'Weight Lifting Exercise Program' OR 'Weight Lifting Exercise Programs' OR 'Weight Lifting Strengthening Program' OR 'Weight Lifting Strengthening Programs' | 91,530 |
| 8 | (#1 OR #2) AND #3 AND (#4 OR #5 OR #6 OR #7) | 651 |

**(Web of Science)**

| Search number | Query | Results |
| --- | --- | --- |
| 1 | TS=( (Anterior Cervical Pains) OR (Anterior Neck Pains) OR (Anterior Neck Pain) OR (Cervical Pain) OR (Cervical Pains) OR (Cervicalgia) OR (Cervicalgias) OR (Cervicodynia) OR (Cervicodynias) OR (Neck Ache) OR (Neck Aches) OR (neck pain) OR (Neck Pains) OR (Neckache) OR (Neckaches) OR (pain, neck) OR (Posterior Cervical Pain) OR (Posterior Cervical Pains) OR (Posterior Neck Pains) OR (Posterior Neck Pain)) | 57,015 |
| 2 | TS=((Nonspecific) OR (non specific) OR (non-specific)) | 680,255 |
| 3 | TS=((Active Stretching) OR (Ballistic Stretching) OR (Continuous Passive Motion Therapy) OR (Continuous Passive Movement Therapy) OR (core stabilisation exercise) OR (core stabilisation training) OR (core stability exercise) OR (core stability training) OR (core stabilization exercise) OR (core stabilization training) OR (CPM Therapies) OR (CPM Therapy) OR (Dynamic Stretching) OR (Isometric Stretching) OR (motion therapy) OR (movement based therapy) OR (movement therapy) OR (Muscle Stretching Exercise) OR (Muscle Stretching Exercises) OR (Passive Stretching) OR (PNF Stretching) OR (PNF Stretching Exercise) OR (PNF Stretching Exercises) OR (PNF Stretchings) OR (Proprioceptive Neuromuscular Facilitation) OR (Proprioceptive Neuromuscular Facilitation (PNF) Stretching) OR (Proprioceptive Neuromuscular Facilitations) OR (Relaxed Stretching) OR (resistance exercise) OR (resistance exercise training) OR (Resistance Training) OR (resistance type exercise) OR (resistance type training) OR (stabilisation exercise) OR (stabilisation training) OR (Stability Training) OR (stabilization exercise) OR (stabilization training) OR (Static Active Stretching) OR (Static Passive Stretching) OR (Static Stretching) OR (Strength Training) OR (strength type exercise) OR (strength type training) OR (stretching exercise) OR (stretching exercises) OR (Weight Bearing Exercise Program) OR (Weight Bearing Exercise Programs) OR (Weight Bearing Strengthening Program) OR (Weight Bearing Strengthening Programs) OR (Weight Lifting Exercise Program) OR (Weight Lifting Exercise Programs) OR (Weight Lifting Strengthening Program) OR (Weight Lifting Strengthening Programs)) | 330,027 |
| 4 | #3 AND #2 AND #1 | 342 |

**Supplementary Table S3** Treatment coding table used in the network meta-analysis

| id | description |
| --- | --- |
| 1 | SOE |
| 2 | PT |
| 3 | NFT |
| 4 | NRT |
| 5 | SFT |
| 6 | SRT |
| 7 | CFT |
| 8 | CRT |
| 9 | NFT+NRT |
| 10 | NFT+NRT+SRT |
| 11 | SRT+NFT |
| 12 | SRT+CFT |
| 13 | NRT+CRT |
| 14 | NFT+SFT |
| 15 | NRT+CRT+SRT |
| 16 | NFT+SFT+NRT |

**Supplementary Table S4** Meta-regression results for VAS

| Parameter | coef | 95% CrI |
| --- | --- | --- |
| Duration | | |
| 1.10 | 2.26728 | [-21.98287; 55.7232] |
| 1.15 | 0.17711 | [-3.57221; 3.8961] |
| 13.8 | 0.88965 | [-4.06066; 6.4473] |
| 14.6 | -0.36927 | [-31.24846; 24.1550] |
| 16.14 | 0.48109 | [-21.25512; 24.6089] |
| 16.4 | -0.25806 | [-4.11119; 3.3893] |
| 4.1 | -2.83766 | [-16.26452; 5.1357] |
| 4.13 | 0.71595 | [-18.23160; 23.4423] |
| 4.2 | 1.65639 | [-28.97622; 57.6340] |
| 4.3 | 3.19515 | [-15.32004; 50.4943] |
| 4.9 | 0.64926 | [-19.27586; 27.4864] |
| 6.11 | 0.76634 | [-4.65146; 6.9934] |
| 6.12 | 1.08364 | [-26.57158; 32.5840] |
| Sample size | | |
| 1.10 | -3.59805 | [-16.4968; 4.2793] |
| 1.15 | 1.07889 | [-2.6946; 5.2005] |
| 13.8 | -1.49676 | [-5.5491; 1.4817] |
| 14.6 | -1.55081 | [-41.7478; 16.0085] |
| 16.14 | 0.84206 | [-11.5767; 20.1650] |
| 16.4 | 0.49163 | [-3.0560; 4.1153] |
| 4.1 | 2.56203 | [-11.3881; 32.1066] |
| 4.13 | 2.02056 | [-17.6637; 50.1379] |
| 4.2 | -0.50263 | [-20.6444; 13.6891] |
| 4.3 | -0.24411 | [-4.5508; 3.1457] |
| 4.9 | 0.78178 | [-9.2880; 13.1099] |
| 6.11 | 0.78130 | [-4.5563; 6.9628] |
| 6.12 | 0.27887 | [-17.4608; 20.0444] |

**Supplementary Table S5** Meta-regression results for NDI

| Parameter | coef | 95% CrI |
| --- | --- | --- |
| Duration | | |
| 10.1 | 3.6237 | [-96.184; 128.542] |
| 13.8 | -1.0398 | [-58.673; 29.977] |
| 2.10 | -6.2030 | [-72.877; 46.215] |
| 2.15 | 4.3376 | [-125.566; 153.932] |
| 2.4 | -1.8908 | [-61.498; 28.869] |
| 4.13 | 18.1345 | [-37.200; 104.046] |
| 4.3 | 0.8578 | [-103.663; 99.147] |
| 4.9 | -14.2604 | [-142.572; 65.434] |
| Sample size | | |
| 10.1 | -18.1670 | [-156.733; 54.651] |
| 13.8 | 7.9182 | [-21.897; 43.086] |
| 2.10 | -6.8132 | [-34.275; 20.438] |
| 2.15 | -43.3107 | [-167.386; 97.613] |
| 2.4 | 0.8561 | [-28.262; 29.571] |
| 4.13 | 18.1765 | [-35.107; 92.081] |
| 4.3 | 0.9113 | [-28.889; 30.411] |
| 4.9 | 5.0397 | [-116.851; 153.002] |

**Supplementary Table S6** Meta-regression results for neck flexion ROM

| Parameter | coef | 95% CrI |
| --- | --- | --- |
| Duration | | |
| 13.8 | 5.226 | [-29.780; 52.15] |
| 2.10 | 2.401 | [-36.031; 55.78] |
| 2.15 | -50.214 | [-204.198; 16.73] |
| 4.13 | -12.622 | [-59.958; 24.11] |
| 4.2 | -8.269 | [-267.656; 146.88] |
| 4.3 | -8.772 | [-82.763; 49.69] |
| 4.9 | -6.174 | [-128.131; 60.11] |
| Sample size | | |
| 13.8 | 0.8269 | [-29.131; 35.21] |
| 2.10 | 8.3160 | [-17.910; 40.68] |
| 2.15 | 3.7014 | [-63.232; 75.46] |
| 4.13 | 4.8557 | [-21.537; 35.46] |
| 4.2 | -3.7506 | [-168.449; 147.91] |
| 4.3 | 13.0636 | [-23.099; 60.21] |
| 4.9 | 8.4794 | [-89.066; 186.77] |

**Supplementary Table S7** Meta-regression results for neck extension ROM

| Parameter | coef | 95% CrI |
| --- | --- | --- |
| Duration | | |
| 13.8 | 8.7615 | [-17.145; 51.449] |
| 2.10 | 6.3128 | [-18.902; 45.812] |
| 2.15 | 18.0799 | [-12.398; 77.104] |
| 4.13 | -8.5955 | [-41.684; 25.814] |
| 4.2 | 0.3717 | [-110.919; 96.066] |
| 4.3 | 29.4960 | [-14.149; 91.354] |
| 4.9 | 4.2352 | [-66.565; 117.269] |
| Sample size | | |
| 13.8 | -1.197 | [-31.1425; 24.27] |
| 2.10 | -4.278 | [-31.3536; 25.17] |
| 2.15 | -5.583 | [-70.4612; 18.06] |
| 4.13 | -2.454 | [-28.4944; 19.69] |
| 4.2 | 8.190 | [-107.7264; 189.70] |
| 4.3 | -1.500 | [-51.0727; 30.69] |
| 4.9 | -17.863 | [-435.4483; 153.58] |

**Supplementary Table S8** Certainty of evidence for VAS

| Comparison | Number of studies | Within-study bias | Reporting bias | Indirectness | Imprecision | Heterogeneity | Incoherence | Confidence rating |
| --- | --- | --- | --- | --- | --- | --- | --- | --- |
| CRT:NRT | 1 | Some concerns | Some concerns | No concerns | Some concerns | Some concerns | No concerns | Very low |
| CRT:NRT+CRT | 1 | Some concerns | Some concerns | No concerns | Some concerns | Some concerns | No concerns | Very low |
| NFT:NFT+NRT | 3 | Some concerns | Some concerns | No concerns | No concerns | Major concerns | No concerns | Low |
| NFT:NRT | 3 | No concerns | Some concerns | No concerns | No concerns | Major concerns | No concerns | Low |
| NFT+NRT:NRT | 2 | No concerns | Some concerns | No concerns | No concerns | Major concerns | No concerns | Low |
| NFT+NRT+SRT:PT | 1 | No concerns | Some concerns | No concerns | Some concerns | Some concerns | No concerns | Low |
| NFT+NRT+SRT:SOE | 1 | No concerns | Some concerns | No concerns | No concerns | Some concerns | No concerns | Low |
| NFT+SFT:NFT+SFT+NRT | 1 | Major concerns | Some concerns | No concerns | No concerns | Some concerns | Major concerns | Very low |
| NFT+SFT:NRT | 1 | Major concerns | Some concerns | No concerns | No concerns | Some concerns | Major concerns | Very low |
| NFT+SFT:SRT | 1 | Some concerns | Some concerns | No concerns | No concerns | Some concerns | Major concerns | Low |
| NFT+SFT+NRT:NRT | 1 | Major concerns | Some concerns | No concerns | Major concerns | No concerns | Major concerns | Very low |
| NRT:NRT+CRT | 2 | Some concerns | Some concerns | No concerns | Some concerns | Some concerns | Major concerns | Very low |
| NRT:NRT+CRT+SRT | 1 | No concerns | Some concerns | No concerns | Some concerns | Some concerns | No concerns | Low |
| NRT:PT | 1 | No concerns | Some concerns | No concerns | Some concerns | Some concerns | No concerns | Low |
| NRT:SOE | 1 | No concerns | Some concerns | No concerns | No concerns | Some concerns | No concerns | Low |
| NRT+CRT+SRT:PT | 1 | No concerns | Some concerns | No concerns | Some concerns | Some concerns | No concerns | Low |
| NRT+CRT+SRT:SOE | 1 | Some concerns | Some concerns | No concerns | Some concerns | No concerns | No concerns | Low |
| SRT:SRT+CFT | 1 | No concerns | Some concerns | No concerns | Some concerns | Some concerns | Major concerns | Low |
| SRT:SRT+NFT | 1 | No concerns | Some concerns | No concerns | Major concerns | No concerns | Major concerns | Very low |
| SRT+CFT:SRT+NFT | 1 | No concerns | Some concerns | No concerns | Some concerns | Some concerns | Major concerns | Low |
| CRT:NFT | 0 | Some concerns | Some concerns | No concerns | Major concerns | No concerns | Major concerns | Very low |
| CRT:NFT+NRT | 0 | Some concerns | Some concerns | No concerns | Some concerns | Some concerns | Major concerns | Very low |
| CRT:NFT+NRT+SRT | 0 | No concerns | Some concerns | No concerns | Major concerns | No concerns | Major concerns | Very low |
| CRT:NFT+SFT | 0 | Major concerns | Some concerns | No concerns | Some concerns | Some concerns | Major concerns | Very low |
| CRT:NFT+SFT+NRT | 0 | Major concerns | Some concerns | No concerns | Major concerns | No concerns | Major concerns | Very low |
| CRT:NRT+CRT+SRT | 0 | Some concerns | Some concerns | No concerns | Some concerns | Some concerns | Major concerns | Very low |
| CRT:PT | 0 | Some concerns | Some concerns | No concerns | Some concerns | Some concerns | Major concerns | Very low |
| CRT:SOE | 0 | Some concerns | Some concerns | No concerns | No concerns | Some concerns | Major concerns | Low |
| CRT:SRT | 0 | Some concerns | Some concerns | No concerns | No concerns | Some concerns | Major concerns | Low |
| CRT:SRT+CFT | 0 | Some concerns | Some concerns | No concerns | No concerns | No concerns | Major concerns | Low |
| CRT:SRT+NFT | 0 | Some concerns | Some concerns | No concerns | No concerns | Some concerns | Major concerns | Low |
| NFT:NFT+NRT+SRT | 0 | No concerns | Some concerns | No concerns | Major concerns | No concerns | Major concerns | Very low |
| NFT:NFT+SFT | 0 | Some concerns | Some concerns | No concerns | Some concerns | No concerns | Major concerns | Low |
| NFT:NFT+SFT+NRT | 0 | Some concerns | Some concerns | No concerns | Major concerns | No concerns | Major concerns | Very low |
| NFT:NRT+CRT | 0 | Some concerns | Some concerns | No concerns | Some concerns | Some concerns | Major concerns | Very low |
| NFT:NRT+CRT+SRT | 0 | No concerns | Some concerns | No concerns | Some concerns | Some concerns | Major concerns | Low |
| NFT:PT | 0 | No concerns | Some concerns | No concerns | Some concerns | Some concerns | Major concerns | Low |
| NFT:SOE | 0 | No concerns | Some concerns | No concerns | No concerns | Some concerns | Major concerns | Low |
| NFT:SRT | 0 | Some concerns | Some concerns | No concerns | No concerns | No concerns | Major concerns | Low |
| NFT:SRT+CFT | 0 | Some concerns | Some concerns | No concerns | No concerns | No concerns | Major concerns | Low |
| NFT:SRT+NFT | 0 | Some concerns | Some concerns | No concerns | No concerns | Some concerns | Major concerns | Low |
| NFT+NRT:NFT+NRT+SRT | 0 | No concerns | Some concerns | No concerns | Major concerns | No concerns | Major concerns | Very low |
| NFT+NRT:NFT+SFT | 0 | Some concerns | Some concerns | No concerns | No concerns | Some concerns | Major concerns | Low |
| NFT+NRT:NFT+SFT+NRT | 0 | Some concerns | Some concerns | No concerns | Major concerns | No concerns | Major concerns | Very low |
| NFT+NRT:NRT+CRT | 0 | Some concerns | Some concerns | No concerns | Some concerns | Some concerns | Major concerns | Very low |
| NFT+NRT:NRT+CRT+SRT | 0 | No concerns | Some concerns | No concerns | Some concerns | Some concerns | Major concerns | Low |
| NFT+NRT:PT | 0 | No concerns | Some concerns | No concerns | Some concerns | Some concerns | Major concerns | Low |
| NFT+NRT:SOE | 0 | No concerns | Some concerns | No concerns | No concerns | Some concerns | Major concerns | Low |
| NFT+NRT:SRT | 0 | Some concerns | Some concerns | No concerns | No concerns | No concerns | Major concerns | Low |
| NFT+NRT:SRT+CFT | 0 | Some concerns | Some concerns | No concerns | No concerns | No concerns | Major concerns | Low |
| NFT+NRT:SRT+NFT | 0 | Some concerns | Some concerns | No concerns | No concerns | No concerns | Major concerns | Low |
| NFT+NRT+SRT:NFT+SFT | 0 | Some concerns | Some concerns | No concerns | Some concerns | No concerns | Major concerns | Low |
| NFT+NRT+SRT:NFT+SFT+NRT | 0 | Some concerns | Some concerns | No concerns | Major concerns | No concerns | Major concerns | Very low |
| NFT+NRT+SRT:NRT | 0 | No concerns | Some concerns | No concerns | Major concerns | No concerns | Major concerns | Very low |
| NFT+NRT+SRT:NRT+CRT | 0 | Some concerns | Some concerns | No concerns | Some concerns | Some concerns | Major concerns | Very low |
| NFT+NRT+SRT:NRT+CRT+SRT | 0 | No concerns | Some concerns | No concerns | Major concerns | No concerns | Major concerns | Very low |
| NFT+NRT+SRT:SRT | 0 | Some concerns | Some concerns | No concerns | No concerns | No concerns | Major concerns | Low |
| NFT+NRT+SRT:SRT+CFT | 0 | Some concerns | Some concerns | No concerns | No concerns | No concerns | Major concerns | Low |
| NFT+NRT+SRT:SRT+NFT | 0 | Some concerns | Some concerns | No concerns | No concerns | Some concerns | Major concerns | Low |
| NFT+SFT:NRT+CRT | 0 | Major concerns | Some concerns | No concerns | Some concerns | Some concerns | Major concerns | Very low |
| NFT+SFT:NRT+CRT+SRT | 0 | Some concerns | Some concerns | No concerns | No concerns | Some concerns | Major concerns | Low |
| NFT+SFT:PT | 0 | Some concerns | Some concerns | No concerns | No concerns | Some concerns | Major concerns | Low |
| NFT+SFT:SOE | 0 | Some concerns | Some concerns | No concerns | No concerns | No concerns | Major concerns | Low |
| NFT+SFT:SRT+CFT | 0 | No concerns | Some concerns | No concerns | No concerns | Some concerns | Major concerns | Low |
| NFT+SFT:SRT+NFT | 0 | No concerns | Some concerns | No concerns | Some concerns | Some concerns | Major concerns | Low |
| NFT+SFT+NRT:NRT+CRT | 0 | Major concerns | Some concerns | No concerns | Some concerns | Some concerns | Major concerns | Very low |
| NFT+SFT+NRT:NRT+CRT+SRT | 0 | Some concerns | Some concerns | No concerns | Major concerns | No concerns | Major concerns | Very low |
| NFT+SFT+NRT:PT | 0 | Some concerns | Some concerns | No concerns | Major concerns | No concerns | Major concerns | Very low |
| NFT+SFT+NRT:SOE | 0 | Some concerns | Some concerns | No concerns | Some concerns | Some concerns | Major concerns | Very low |
| NFT+SFT+NRT:SRT | 0 | Major concerns | Some concerns | No concerns | No concerns | No concerns | Major concerns | Very low |
| NFT+SFT+NRT:SRT+CFT | 0 | Some concerns | Some concerns | No concerns | No concerns | No concerns | Major concerns | Low |
| NFT+SFT+NRT:SRT+NFT | 0 | Some concerns | Some concerns | No concerns | No concerns | No concerns | Major concerns | Low |
| NRT:SRT | 0 | Major concerns | Some concerns | No concerns | No concerns | No concerns | Major concerns | Very low |
| NRT:SRT+CFT | 0 | Some concerns | Some concerns | No concerns | No concerns | No concerns | Major concerns | Low |
| NRT:SRT+NFT | 0 | Some concerns | Some concerns | No concerns | No concerns | No concerns | Major concerns | Low |
| NRT+CRT:NRT+CRT+SRT | 0 | Some concerns | Some concerns | No concerns | Some concerns | Some concerns | Major concerns | Very low |
| NRT+CRT:PT | 0 | Some concerns | Some concerns | No concerns | Some concerns | No concerns | Major concerns | Low |
| NRT+CRT:SOE | 0 | Some concerns | Some concerns | No concerns | No concerns | Some concerns | Major concerns | Low |
| NRT+CRT:SRT | 0 | Major concerns | Some concerns | No concerns | No concerns | Some concerns | Major concerns | Very low |
| NRT+CRT:SRT+CFT | 0 | Some concerns | Some concerns | No concerns | No concerns | No concerns | Major concerns | Low |
| NRT+CRT:SRT+NFT | 0 | Some concerns | Some concerns | No concerns | No concerns | Some concerns | Major concerns | Low |
| NRT+CRT+SRT:SRT | 0 | Some concerns | Some concerns | No concerns | No concerns | No concerns | Major concerns | Low |
| NRT+CRT+SRT:SRT+CFT | 0 | Some concerns | Some concerns | No concerns | No concerns | No concerns | Major concerns | Low |
| NRT+CRT+SRT:SRT+NFT | 0 | Some concerns | Some concerns | No concerns | No concerns | No concerns | Major concerns | Low |
| PT:SOE | 0 | No concerns | Some concerns | No concerns | Some concerns | Some concerns | Major concerns | Low |
| PT:SRT | 0 | Some concerns | Some concerns | No concerns | No concerns | No concerns | Major concerns | Low |
| PT:SRT+CFT | 0 | Some concerns | Some concerns | No concerns | No concerns | No concerns | Major concerns | Low |
| PT:SRT+NFT | 0 | Some concerns | Some concerns | No concerns | No concerns | No concerns | Major concerns | Low |
| SOE:SRT | 0 | Some concerns | Some concerns | No concerns | No concerns | No concerns | Major concerns | Low |
| SOE:SRT+CFT | 0 | Some concerns | Some concerns | No concerns | No concerns | No concerns | Major concerns | Low |
| SOE:SRT+NFT | 0 | Some concerns | Some concerns | No concerns | No concerns | No concerns | Major concerns | Low |

**Supplementary Table S9** Certainty of evidence for NDI

| Comparison | Number of studies | Within-study bias | Reporting bias | Indirectness | Imprecision | Heterogeneity | Incoherence | Confidence rating |
| --- | --- | --- | --- | --- | --- | --- | --- | --- |
| CRT:NRT | 1 | Some concerns | Some concerns | No concerns | Major concerns | No concerns | No concerns | Low |
| CRT:NRT+CRT | 1 | Some concerns | Some concerns | No concerns | Major concerns | No concerns | No concerns | Low |
| NFT:NFT+NRT | 2 | Some concerns | Some concerns | No concerns | Major concerns | No concerns | No concerns | Low |
| NFT:NRT | 3 | No concerns | Some concerns | No concerns | Major concerns | No concerns | No concerns | Low |
| NFT+NRT:NRT | 2 | Some concerns | Some concerns | No concerns | Major concerns | No concerns | No concerns | Low |
| NFT+NRT+SRT:PT | 1 | No concerns | Some concerns | No concerns | Major concerns | No concerns | Major concerns | Very low |
| NFT+NRT+SRT:SOE | 1 | No concerns | Some concerns | No concerns | Major concerns | No concerns | Major concerns | Very low |
| NRT:NRT+CRT | 2 | Some concerns | Some concerns | No concerns | Major concerns | No concerns | Major concerns | Very low |
| NRT:NRT+CRT+SRT | 1 | No concerns | Some concerns | No concerns | Major concerns | No concerns | No concerns | Low |
| NRT:PT | 1 | No concerns | Some concerns | No concerns | Major concerns | No concerns | No concerns | Low |
| NRT+CRT+SRT:PT | 1 | No concerns | Some concerns | No concerns | Major concerns | No concerns | No concerns | Low |
| NRT+CRT+SRT:SOE | 1 | Some concerns | Some concerns | No concerns | Major concerns | No concerns | Major concerns | Very low |
| CRT:NFT | 0 | Some concerns | Some concerns | No concerns | Major concerns | No concerns | Major concerns | Very low |
| CRT:NFT+NRT | 0 | Some concerns | Some concerns | No concerns | Major concerns | No concerns | Major concerns | Very low |
| CRT:NFT+NRT+SRT | 0 | Some concerns | Some concerns | No concerns | Major concerns | No concerns | Major concerns | Very low |
| CRT:NRT+CRT+SRT | 0 | Some concerns | Some concerns | No concerns | Major concerns | No concerns | Major concerns | Very low |
| CRT:PT | 0 | Some concerns | Some concerns | No concerns | Major concerns | No concerns | Major concerns | Very low |
| CRT:SOE | 0 | Some concerns | Some concerns | No concerns | Major concerns | No concerns | Major concerns | Very low |
| NFT:NFT+NRT+SRT | 0 | No concerns | Some concerns | No concerns | Major concerns | No concerns | Major concerns | Very low |
| NFT:NRT+CRT | 0 | Some concerns | Some concerns | No concerns | Major concerns | No concerns | Major concerns | Very low |
| NFT:NRT+CRT+SRT | 0 | No concerns | Some concerns | No concerns | Major concerns | No concerns | Major concerns | Very low |
| NFT:PT | 0 | No concerns | Some concerns | No concerns | Major concerns | No concerns | Major concerns | Very low |
| NFT:SOE | 0 | Some concerns | Some concerns | No concerns | Major concerns | No concerns | Major concerns | Very low |
| NFT+NRT:NFT+NRT+SRT | 0 | No concerns | Some concerns | No concerns | Major concerns | No concerns | Major concerns | Very low |
| NFT+NRT:NRT+CRT | 0 | Some concerns | Some concerns | No concerns | Major concerns | No concerns | Major concerns | Very low |
| NFT+NRT:NRT+CRT+SRT | 0 | No concerns | Some concerns | No concerns | Major concerns | No concerns | Major concerns | Very low |
| NFT+NRT:PT | 0 | No concerns | Some concerns | No concerns | Major concerns | No concerns | Major concerns | Very low |
| NFT+NRT:SOE | 0 | Some concerns | Some concerns | No concerns | Major concerns | No concerns | Major concerns | Very low |
| NFT+NRT+SRT:NRT | 0 | No concerns | Some concerns | No concerns | Major concerns | No concerns | Major concerns | Very low |
| NFT+NRT+SRT:NRT+CRT | 0 | Some concerns | Some concerns | No concerns | Major concerns | No concerns | Major concerns | Very low |
| NFT+NRT+SRT:NRT+CRT+SRT | 0 | Some concerns | Some concerns | No concerns | Major concerns | No concerns | Major concerns | Very low |
| NRT:SOE | 0 | Some concerns | Some concerns | No concerns | Major concerns | No concerns | Major concerns | Very low |
| NRT+CRT:NRT+CRT+SRT | 0 | Some concerns | Some concerns | No concerns | Major concerns | No concerns | Major concerns | Very low |
| NRT+CRT:PT | 0 | Some concerns | Some concerns | No concerns | Major concerns | No concerns | Major concerns | Very low |
| NRT+CRT:SOE | 0 | Some concerns | Some concerns | No concerns | Major concerns | No concerns | Major concerns | Very low |
| PT:SOE | 0 | No concerns | Some concerns | No concerns | Major concerns | No concerns | Major concerns | Very low |

**Supplementary Table S10** Certainty of evidence for Neck flexion

| Comparison | Number of studies | Within-study bias | Reporting bias | Indirectness | Imprecision | Heterogeneity | Incoherence | Confidence rating |
| --- | --- | --- | --- | --- | --- | --- | --- | --- |
| CRT:NRT | 1 | Some concerns | Some concerns | No concerns | Major concerns | No concerns | No concerns | Low |
| CRT:NRT+CRT | 1 | Some concerns | Some concerns | No concerns | Major concerns | No concerns | No concerns | Low |
| NFT:NFT+NRT | 1 | No concerns | Some concerns | No concerns | No concerns | Major concerns | No concerns | Low |
| NFT:NRT | 2 | No concerns | Some concerns | No concerns | Major concerns | No concerns | No concerns | Low |
| NFT+NRT:NRT | 2 | No concerns | Some concerns | No concerns | No concerns | Major concerns | No concerns | Low |
| NFT+NRT+SRT:PT | 1 | No concerns | Some concerns | No concerns | Major concerns | No concerns | Major concerns | Very low |
| NRT:NRT+CRT | 2 | Some concerns | Some concerns | No concerns | Some concerns | Some concerns | Major concerns | Very low |
| NRT:NRT+CRT+SRT | 1 | No concerns | Some concerns | No concerns | Major concerns | No concerns | Major concerns | Very low |
| NRT:PT | 1 | No concerns | Some concerns | No concerns | Some concerns | Some concerns | Major concerns | Low |
| NRT+CRT+SRT:PT | 1 | No concerns | Some concerns | No concerns | Major concerns | No concerns | Major concerns | Very low |
| CRT:NFT | 0 | Some concerns | Some concerns | No concerns | Major concerns | No concerns | Major concerns | Very low |
| CRT:NFT+NRT | 0 | Some concerns | Some concerns | No concerns | Some concerns | Some concerns | Major concerns | Very low |
| CRT:NFT+NRT+SRT | 0 | No concerns | Some concerns | No concerns | Major concerns | No concerns | Major concerns | Very low |
| CRT:NRT+CRT+SRT | 0 | Some concerns | Some concerns | No concerns | Major concerns | No concerns | Major concerns | Very low |
| CRT:PT | 0 | Some concerns | Some concerns | No concerns | Major concerns | No concerns | Major concerns | Very low |
| NFT:NFT+NRT+SRT | 0 | No concerns | Some concerns | No concerns | Major concerns | No concerns | Major concerns | Very low |
| NFT:NRT+CRT | 0 | Some concerns | Some concerns | No concerns | Major concerns | No concerns | Major concerns | Very low |
| NFT:NRT+CRT+SRT | 0 | No concerns | Some concerns | No concerns | Major concerns | No concerns | Major concerns | Very low |
| NFT:PT | 0 | No concerns | Some concerns | No concerns | Major concerns | No concerns | Major concerns | Very low |
| NFT+NRT:NFT+NRT+SRT | 0 | No concerns | Some concerns | No concerns | No concerns | Major concerns | Major concerns | Very low |
| NFT+NRT:NRT+CRT | 0 | Some concerns | Some concerns | No concerns | Some concerns | Some concerns | Major concerns | Very low |
| NFT+NRT:NRT+CRT+SRT | 0 | No concerns | Some concerns | No concerns | No concerns | Major concerns | Major concerns | Very low |
| NFT+NRT:PT | 0 | No concerns | Some concerns | No concerns | No concerns | Some concerns | Major concerns | Low |
| NFT+NRT+SRT:NRT | 0 | No concerns | Some concerns | No concerns | Major concerns | No concerns | Major concerns | Very low |
| NFT+NRT+SRT:NRT+CRT | 0 | No concerns | Some concerns | No concerns | Some concerns | Some concerns | Major concerns | Low |
| NFT+NRT+SRT:NRT+CRT+SRT | 0 | No concerns | Some concerns | No concerns | Major concerns | No concerns | Major concerns | Very low |
| NRT+CRT:NRT+CRT+SRT | 0 | Some concerns | Some concerns | No concerns | Major concerns | No concerns | Major concerns | Very low |
| NRT+CRT:PT | 0 | Some concerns | Some concerns | No concerns | No concerns | Major concerns | Major concerns | Very low |

**Supplementary Table S11** Certainty of evidence for Neck extension

| Comparison | Number of studies | Within-study bias | Reporting bias | Indirectness | Imprecision | Heterogeneity | Incoherence | Confidence rating |
| --- | --- | --- | --- | --- | --- | --- | --- | --- |
| CRT:NRT | 1 | Some concerns | Some concerns | No concerns | Major concerns | No concerns | Some concerns | Low |
| CRT:NRT+CRT | 1 | Some concerns | Some concerns | No concerns | Major concerns | No concerns | Some concerns | Low |
| NFT:NFT+NRT | 1 | No concerns | Some concerns | No concerns | Some concerns | Some concerns | No concerns | Low |
| NFT:NRT | 2 | No concerns | Some concerns | No concerns | Major concerns | No concerns | No concerns | Low |
| NFT+NRT:NRT | 2 | No concerns | Some concerns | No concerns | No concerns | Major concerns | No concerns | Low |
| NFT+NRT+SRT:PT | 1 | No concerns | Some concerns | No concerns | Major concerns | No concerns | Some concerns | Low |
| NRT:NRT+CRT | 1 | Some concerns | Some concerns | No concerns | Major concerns | No concerns | Some concerns | Low |
| NRT:NRT+CRT+SRT | 1 | No concerns | Some concerns | No concerns | Major concerns | No concerns | Some concerns | Low |
| NRT:PT | 1 | No concerns | Some concerns | No concerns | Some concerns | Some concerns | Some concerns | Very low |
| NRT+CRT+SRT:PT | 1 | No concerns | Some concerns | No concerns | Major concerns | No concerns | Some concerns | Low |
| CRT:NFT | 0 | No concerns | Some concerns | No concerns | Major concerns | No concerns | Some concerns | Low |
| CRT:NFT+NRT | 0 | Some concerns | Some concerns | No concerns | Some concerns | Some concerns | Some concerns | Very low |
| CRT:NFT+NRT+SRT | 0 | No concerns | Some concerns | No concerns | Major concerns | No concerns | Some concerns | Low |
| CRT:NRT+CRT+SRT | 0 | Some concerns | Some concerns | No concerns | Major concerns | No concerns | Some concerns | Low |
| CRT:PT | 0 | No concerns | Some concerns | No concerns | Major concerns | No concerns | Some concerns | Low |
| NFT:NFT+NRT+SRT | 0 | No concerns | Some concerns | No concerns | Major concerns | No concerns | Some concerns | Low |
| NFT:NRT+CRT | 0 | No concerns | Some concerns | No concerns | Major concerns | No concerns | Some concerns | Low |
| NFT:NRT+CRT+SRT | 0 | No concerns | Some concerns | No concerns | Major concerns | No concerns | Some concerns | Low |
| NFT:PT | 0 | No concerns | Some concerns | No concerns | Some concerns | Some concerns | Some concerns | Very low |
| NFT+NRT:NFT+NRT+SRT | 0 | No concerns | Some concerns | No concerns | No concerns | Major concerns | Some concerns | Low |
| NFT+NRT:NRT+CRT | 0 | Some concerns | Some concerns | No concerns | Some concerns | Some concerns | Some concerns | Very low |
| NFT+NRT:NRT+CRT+SRT | 0 | No concerns | Some concerns | No concerns | Some concerns | Some concerns | Some concerns | Very low |
| NFT+NRT:PT | 0 | No concerns | Some concerns | No concerns | No concerns | Major concerns | Some concerns | Low |
| NFT+NRT+SRT:NRT | 0 | No concerns | Some concerns | No concerns | Major concerns | No concerns | Some concerns | Low |
| NFT+NRT+SRT:NRT+CRT | 0 | No concerns | Some concerns | No concerns | Major concerns | No concerns | Some concerns | Low |
| NFT+NRT+SRT:NRT+CRT+SRT | 0 | No concerns | Some concerns | No concerns | Major concerns | No concerns | Some concerns | Low |
| NRT+CRT:NRT+CRT+SRT | 0 | Some concerns | Some concerns | No concerns | Major concerns | No concerns | Some concerns | Low |
| NRT+CRT:PT | 0 | No concerns | Some concerns | No concerns | Major concerns | No concerns | Some concerns | Low |

**Supplementary Table S12** Certainty of evidence for Neck left lateral flexion

| Comparison | Number of studies | Within-study bias | Reporting bias | Indirectness | Imprecision | Heterogeneity | Incoherence | Confidence rating |
| --- | --- | --- | --- | --- | --- | --- | --- | --- |
| CRT:NRT | 1 | Some concerns | Some concerns | No concerns | Major concerns | No concerns | No concerns | Low |
| CRT:NRT+CRT | 1 | Some concerns | Some concerns | No concerns | Major concerns | No concerns | No concerns | Low |
| NFT:NFT+NRT | 1 | No concerns | Some concerns | No concerns | No concerns | Major concerns | No concerns | Low |
| NFT:NRT | 2 | No concerns | Some concerns | No concerns | Major concerns | No concerns | No concerns | Low |
| NFT+NRT:NRT | 2 | No concerns | Some concerns | No concerns | No concerns | Major concerns | No concerns | Low |
| NFT+NRT+SRT:PT | 1 | No concerns | Some concerns | No concerns | Major concerns | No concerns | No concerns | Low |
| NRT:NRT+CRT | 1 | Some concerns | Some concerns | No concerns | Major concerns | No concerns | No concerns | Low |
| NRT:NRT+CRT+SRT | 1 | No concerns | Some concerns | No concerns | Some concerns | Some concerns | No concerns | Low |
| NRT:PT | 1 | No concerns | Some concerns | No concerns | Major concerns | No concerns | No concerns | Low |
| NRT+CRT+SRT:PT | 1 | No concerns | Some concerns | No concerns | No concerns | Major concerns | No concerns | Low |
| CRT:NFT | 0 | Some concerns | Some concerns | No concerns | Major concerns | No concerns | No concerns | Low |
| CRT:NFT+NRT | 0 | Some concerns | Some concerns | No concerns | Some concerns | Some concerns | No concerns | Very low |
| CRT:NFT+NRT+SRT | 0 | No concerns | Some concerns | No concerns | Major concerns | No concerns | No concerns | Low |
| CRT:NRT+CRT+SRT | 0 | Some concerns | Some concerns | No concerns | Major concerns | No concerns | No concerns | Low |
| CRT:PT | 0 | No concerns | Some concerns | No concerns | Major concerns | No concerns | No concerns | Low |
| NFT:NFT+NRT+SRT | 0 | No concerns | Some concerns | No concerns | Major concerns | No concerns | No concerns | Low |
| NFT:NRT+CRT | 0 | Some concerns | Some concerns | No concerns | Major concerns | No concerns | No concerns | Low |
| NFT:NRT+CRT+SRT | 0 | No concerns | Some concerns | No concerns | Major concerns | No concerns | No concerns | Low |
| NFT:PT | 0 | No concerns | Some concerns | No concerns | Major concerns | No concerns | No concerns | Low |
| NFT+NRT:NFT+NRT+SRT | 0 | No concerns | Some concerns | No concerns | No concerns | Major concerns | No concerns | Low |
| NFT+NRT:NRT+CRT | 0 | Some concerns | Some concerns | No concerns | Major concerns | No concerns | No concerns | Low |
| NFT+NRT:NRT+CRT+SRT | 0 | No concerns | Some concerns | No concerns | Major concerns | No concerns | No concerns | Low |
| NFT+NRT:PT | 0 | No concerns | Some concerns | No concerns | No concerns | Major concerns | No concerns | Low |
| NFT+NRT+SRT:NRT | 0 | No concerns | Some concerns | No concerns | Major concerns | No concerns | No concerns | Low |
| NFT+NRT+SRT:NRT+CRT | 0 | No concerns | Some concerns | No concerns | Some concerns | Some concerns | No concerns | Low |
| NFT+NRT+SRT:NRT+CRT+SRT | 0 | No concerns | Some concerns | No concerns | No concerns | Major concerns | No concerns | Low |
| NRT+CRT:NRT+CRT+SRT | 0 | Some concerns | Some concerns | No concerns | Major concerns | No concerns | No concerns | Low |
| NRT+CRT:PT | 0 | No concerns | Some concerns | No concerns | Major concerns | No concerns | No concerns | Low |

**Supplementary Table S13** Certainty of evidence for Neck right lateral flexion

| Comparison | Number of studies | Within-study bias | Reporting bias | Indirectness | Imprecision | Heterogeneity | Incoherence | Confidence rating |
| --- | --- | --- | --- | --- | --- | --- | --- | --- |
| CRT:NRT | 1 | Some concerns | Some concerns | No concerns | Major concerns | No concerns | Some concerns | Low |
| CRT:NRT+CRT | 1 | Some concerns | Some concerns | No concerns | Major concerns | No concerns | Some concerns | Low |
| NFT:NFT+NRT | 1 | No concerns | Some concerns | No concerns | No concerns | Major concerns | No concerns | Low |
| NFT:NRT | 2 | No concerns | Some concerns | No concerns | Major concerns | No concerns | No concerns | Low |
| NFT+NRT:NRT | 2 | No concerns | Some concerns | No concerns | No concerns | Major concerns | No concerns | Low |
| NFT+NRT+SRT:PT | 1 | No concerns | Some concerns | No concerns | Major concerns | No concerns | Some concerns | Low |
| NRT:NRT+CRT | 1 | Some concerns | Some concerns | No concerns | Major concerns | No concerns | Some concerns | Low |
| NRT:NRT+CRT+SRT | 1 | No concerns | Some concerns | No concerns | Some concerns | Some concerns | Some concerns | Very low |
| NRT:PT | 1 | No concerns | Some concerns | No concerns | Major concerns | No concerns | Some concerns | Low |
| NRT+CRT+SRT:PT | 1 | No concerns | Some concerns | No concerns | No concerns | Major concerns | Some concerns | Low |
| CRT:NFT | 0 | Some concerns | Some concerns | No concerns | Major concerns | No concerns | Some concerns | Low |
| CRT:NFT+NRT | 0 | Some concerns | Some concerns | No concerns | Some concerns | Some concerns | Some concerns | Very low |
| CRT:NFT+NRT+SRT | 0 | No concerns | Some concerns | No concerns | Major concerns | No concerns | Some concerns | Low |
| CRT:NRT+CRT+SRT | 0 | Some concerns | Some concerns | No concerns | Major concerns | No concerns | Some concerns | Low |
| CRT:PT | 0 | Some concerns | Some concerns | No concerns | Major concerns | No concerns | Some concerns | Low |
| NFT:NFT+NRT+SRT | 0 | No concerns | Some concerns | No concerns | Major concerns | No concerns | Some concerns | Low |
| NFT:NRT+CRT | 0 | Some concerns | Some concerns | No concerns | Major concerns | No concerns | Some concerns | Low |
| NFT:NRT+CRT+SRT | 0 | No concerns | Some concerns | No concerns | Major concerns | No concerns | Some concerns | Low |
| NFT:PT | 0 | No concerns | Some concerns | No concerns | Major concerns | No concerns | Some concerns | Low |
| NFT+NRT:NFT+NRT+SRT | 0 | No concerns | Some concerns | No concerns | No concerns | Major concerns | Some concerns | Low |
| NFT+NRT:NRT+CRT | 0 | Some concerns | Some concerns | No concerns | Some concerns | Some concerns | Some concerns | Very low |
| NFT+NRT:NRT+CRT+SRT | 0 | No concerns | Some concerns | No concerns | Major concerns | No concerns | Some concerns | Low |
| NFT+NRT:PT | 0 | No concerns | Some concerns | No concerns | No concerns | Major concerns | Some concerns | Low |
| NFT+NRT+SRT:NRT | 0 | No concerns | Some concerns | No concerns | Major concerns | No concerns | Some concerns | Low |
| NFT+NRT+SRT:NRT+CRT | 0 | No concerns | Some concerns | No concerns | Major concerns | No concerns | Some concerns | Low |
| NFT+NRT+SRT:NRT+CRT+SRT | 0 | No concerns | Some concerns | No concerns | No concerns | Major concerns | Some concerns | Low |
| NRT+CRT:NRT+CRT+SRT | 0 | Some concerns | Some concerns | No concerns | Major concerns | No concerns | Some concerns | Low |
| NRT+CRT:PT | 0 | Some concerns | Some concerns | No concerns | Major concerns | No concerns | Some concerns | Low |

**Supplementary Table S14** Certainty of evidence for Neck left rotation

| Comparison | Number of studies | Within-study bias | Reporting bias | Indirectness | Imprecision | Heterogeneity | Incoherence | Confidence rating |
| --- | --- | --- | --- | --- | --- | --- | --- | --- |
| CRT:NRT | 1 | Some concerns | Some concerns | No concerns | Major concerns | No concerns | No concerns | Low |
| CRT:NRT+CRT | 1 | Some concerns | Some concerns | No concerns | Major concerns | No concerns | No concerns | Low |
| NFT:NFT+NRT | 1 | No concerns | Some concerns | No concerns | No concerns | Major concerns | No concerns | Low |
| NFT:NRT | 2 | No concerns | Some concerns | No concerns | Major concerns | No concerns | No concerns | Low |
| NFT+NRT:NRT | 2 | No concerns | Some concerns | No concerns | No concerns | Major concerns | No concerns | Low |
| NFT+NRT+SRT:PT | 1 | No concerns | Some concerns | No concerns | Major concerns | No concerns | No concerns | Low |
| NRT:NRT+CRT | 1 | Some concerns | Some concerns | No concerns | Major concerns | No concerns | No concerns | Low |
| NRT:NRT+CRT+SRT | 1 | No concerns | Some concerns | No concerns | Major concerns | No concerns | No concerns | Low |
| NRT:PT | 1 | No concerns | Some concerns | No concerns | No concerns | Major concerns | No concerns | Low |
| NRT+CRT+SRT:PT | 1 | No concerns | Some concerns | No concerns | No concerns | Major concerns | No concerns | Low |
| CRT:NFT | 0 | Some concerns | Some concerns | No concerns | Major concerns | No concerns | No concerns | Low |
| CRT:NFT+NRT | 0 | Some concerns | Some concerns | No concerns | No concerns | Major concerns | No concerns | Low |
| CRT:NFT+NRT+SRT | 0 | No concerns | Some concerns | No concerns | No concerns | Major concerns | No concerns | Low |
| CRT:NRT+CRT+SRT | 0 | Some concerns | Some concerns | No concerns | Major concerns | No concerns | No concerns | Low |
| CRT:PT | 0 | Some concerns | Some concerns | No concerns | Some concerns | Some concerns | No concerns | Very low |
| NFT:NFT+NRT+SRT | 0 | No concerns | Some concerns | No concerns | No concerns | Major concerns | No concerns | Low |
| NFT:NRT+CRT | 0 | Some concerns | Some concerns | No concerns | Major concerns | No concerns | No concerns | Low |
| NFT:NRT+CRT+SRT | 0 | No concerns | Some concerns | No concerns | Major concerns | No concerns | No concerns | Low |
| NFT:PT | 0 | No concerns | Some concerns | No concerns | No concerns | Major concerns | No concerns | Low |
| NFT+NRT:NFT+NRT+SRT | 0 | No concerns | Some concerns | No concerns | No concerns | No concerns | No concerns | Moderate |
| NFT+NRT:NRT+CRT | 0 | Some concerns | Some concerns | No concerns | No concerns | Major concerns | No concerns | Low |
| NFT+NRT:NRT+CRT+SRT | 0 | No concerns | Some concerns | No concerns | No concerns | Major concerns | No concerns | Low |
| NFT+NRT:PT | 0 | No concerns | Some concerns | No concerns | No concerns | No concerns | No concerns | Moderate |
| NFT+NRT+SRT:NRT | 0 | No concerns | Some concerns | No concerns | No concerns | Major concerns | No concerns | Low |
| NFT+NRT+SRT:NRT+CRT | 0 | No concerns | Some concerns | No concerns | No concerns | Major concerns | No concerns | Low |
| NFT+NRT+SRT:NRT+CRT+SRT | 0 | No concerns | Some concerns | No concerns | No concerns | Major concerns | No concerns | Low |
| NRT+CRT:NRT+CRT+SRT | 0 | Some concerns | Some concerns | No concerns | Major concerns | No concerns | No concerns | Low |
| NRT+CRT:PT | 0 | Some concerns | Some concerns | No concerns | Some concerns | Some concerns | No concerns | Very low |

**Supplementary Table S15** Certainty of evidence for Neck right rotation

| Comparison | Number of studies | Within-study bias | Reporting bias | Indirectness | Imprecision | Heterogeneity | Incoherence | Confidence rating |
| --- | --- | --- | --- | --- | --- | --- | --- | --- |
| CRT:NRT | 1 | Some concerns | Some concerns | No concerns | Major concerns | No concerns | Major concerns | Very low |
| CRT:NRT+CRT | 1 | Some concerns | Some concerns | No concerns | Major concerns | No concerns | Major concerns | Very low |
| NFT:NFT+NRT | 1 | No concerns | Some concerns | No concerns | No concerns | Major concerns | No concerns | Low |
| NFT:NRT | 2 | No concerns | Some concerns | No concerns | Major concerns | No concerns | No concerns | Low |
| NFT+NRT:NRT | 2 | No concerns | Some concerns | No concerns | No concerns | Major concerns | No concerns | Low |
| NFT+NRT+SRT:PT | 1 | No concerns | Some concerns | No concerns | Major concerns | No concerns | Major concerns | Very low |
| NRT:NRT+CRT | 1 | Some concerns | Some concerns | No concerns | Major concerns | No concerns | Major concerns | Very low |
| NRT:NRT+CRT+SRT | 1 | No concerns | Some concerns | No concerns | Major concerns | No concerns | Major concerns | Very low |
| NRT:PT | 1 | No concerns | Some concerns | No concerns | Major concerns | No concerns | Major concerns | Very low |
| NRT+CRT+SRT:PT | 1 | No concerns | Some concerns | No concerns | Some concerns | Some concerns | Major concerns | Low |
| CRT:NFT | 0 | Some concerns | Some concerns | No concerns | Major concerns | No concerns | Major concerns | Very low |
| CRT:NFT+NRT | 0 | Some concerns | Some concerns | No concerns | No concerns | Major concerns | Major concerns | Very low |
| CRT:NFT+NRT+SRT | 0 | No concerns | Some concerns | No concerns | Major concerns | No concerns | Major concerns | Very low |
| CRT:NRT+CRT+SRT | 0 | Some concerns | Some concerns | No concerns | Major concerns | No concerns | Major concerns | Very low |
| CRT:PT | 0 | Some concerns | Some concerns | No concerns | Major concerns | No concerns | Major concerns | Very low |
| NFT:NFT+NRT+SRT | 0 | No concerns | Some concerns | No concerns | Major concerns | No concerns | Major concerns | Very low |
| NFT:NRT+CRT | 0 | Some concerns | Some concerns | No concerns | Major concerns | No concerns | Major concerns | Very low |
| NFT:NRT+CRT+SRT | 0 | No concerns | Some concerns | No concerns | Major concerns | No concerns | Major concerns | Very low |
| NFT:PT | 0 | No concerns | Some concerns | No concerns | Major concerns | No concerns | Major concerns | Very low |
| NFT+NRT:NFT+NRT+SRT | 0 | No concerns | Some concerns | No concerns | No concerns | Major concerns | Major concerns | Very low |
| NFT+NRT:NRT+CRT | 0 | Some concerns | Some concerns | No concerns | Some concerns | Some concerns | Major concerns | Very low |
| NFT+NRT:NRT+CRT+SRT | 0 | No concerns | Some concerns | No concerns | Some concerns | Some concerns | Major concerns | Low |
| NFT+NRT:PT | 0 | No concerns | Some concerns | No concerns | No concerns | Major concerns | Major concerns | Very low |
| NFT+NRT+SRT:NRT | 0 | No concerns | Some concerns | No concerns | Major concerns | No concerns | Major concerns | Very low |
| NFT+NRT+SRT:NRT+CRT | 0 | No concerns | Some concerns | No concerns | Major concerns | No concerns | Major concerns | Very low |
| NFT+NRT+SRT:NRT+CRT+SRT | 0 | No concerns | Some concerns | No concerns | Some concerns | Some concerns | Major concerns | Low |
| NRT+CRT:NRT+CRT+SRT | 0 | Some concerns | Some concerns | No concerns | Major concerns | No concerns | Major concerns | Very low |
| NRT+CRT:PT | 0 | Some concerns | Some concerns | No concerns | Major concerns | No concerns | Major concerns | Very low |

**A**

**B**

**C**

**D**

**E**

**F**

**G**

**H**

**Supplementary Figure S1** Fitting results of PSRF. (Note: A: VAS; B: NDI; C: Neck flexion ROM; D: Neck extension ROM; E: Neck left lateral flexion ROM; F: Neck right lateral flexion ROM; G: Neck left rotation ROM; H: Neck right rotation ROM.)

A

B

C

D

E

F

G

**Supplementary Figure S2** MCMC convergence diagnostics.(Note: A: VAS; B: NDI; C: Neck flexion ROM; D: Neck extension ROM; E: Neck left lateral flexion ROM; F: Neck right lateral flexion ROM; G: Neck left rotation ROM; H: Neck right rotation ROM.)

**A**

**B**

**C**

**D**

**E**

**F**

**G**

**H**

**Supplementary Figure S3** Fitting results of PSRF. (Note: A: VAS; B: NDI; C: Neck flexion ROM; D: Neck extension ROM; E: Neck left lateral flexion ROM; F: Neck right lateral flexion ROM; G: Neck left rotation ROM; H: Neck right rotation ROM.)
